# The YAP/HIF-1α/miR-182/EGR2 axis is implicated in asthma severity through the control of Th17 cell differentiation

**DOI:** 10.1186/s13578-021-00560-1

**Published:** 2021-05-12

**Authors:** Jing Zhou, Ning Zhang, Wei Zhang, Caiju Lu, Fei Xu

**Affiliations:** 1 Department of Respiratory Medicine, The First Affiliated Hospital of Nanchang University, No. 17, Yongwai Street, Donghu District, Nanchang, 330006 People’s Republic of China; 2 Department of Imaging, The First Affiliated Hospital of Nanchang University, Nanchang, 330006 People’s Republic of China

**Keywords:** YAP, HIF-1α, MiR-182, EGR2, Th17 cells, Differentiation, Asthma, Dyslipidemia

## Abstract

**Background:**

Asthma is a heterogeneous chronic inflammatory disease of the airway, involving reversible airflow limitation and airway remodeling. T helper 17 (Th17) cells play an important role in the pathogenesis of allergic asthma. However, there is limited understanding of the signaling pathways controlling Th17 cell differentiation in asthma. The aim of this study was to investigate if the Yes-associated protein (YAP)/hypoxia inducible factor-1α (HIF-1α)/microRNA-182 (miR-182)/early growth response 2 (EGR2) axis is involved in mediating Th17 cell differentiation and disease severity in asthma.

**Methods:**

The study included 29 pediatric patients with asthma, 22 healthy volunteers, ovalbumin-induced murine asthma models, and mouse naive CD4^+^ T cells. The subpopulation of Th17 cells was examined by flow cytometry. The levels of interleukin-17A were determined by enzyme linked immunosorbent assay. Chromatin immunoprecipitation-quantitative polymerase chain reaction assays and dual-luciferase reporter gene assays were performed to examine interactions between HIF-1α and miR-182, and between miR-182 and EGR2.

**Results:**

YAP, HIF-1α, and miR-182 were upregulated but EGR2 was downregulated in human and mouse peripheral blood mononuclear cells from the asthma group. Abundant expression of YAP and HIF-1α promoted miR-182 expression and then inhibited EGR2, a target of miR-182, thus enhancing Th17 differentiation and deteriorating asthma and lipid metabolism dysfunction. In addition, in vivo overexpression of EGR2 countered the promoting effect of the YAP/HIF-1α/miR-182 axis on asthma and lipid metabolism dysfunction.

**Conclusion:**

These results indicate that activation of the YAP/HIF-1α/miR-182/EGR2 axis may promote Th17 cell differentiation, exacerbate asthma development, and aggravate lipid metabolism dysfunction, thus suggesting a potential therapeutic target for asthma.

**Supplementary Information:**

The online version contains supplementary material available at 10.1186/s13578-021-00560-1.

## Background

Asthma is a frequently occurring disease of the airways, affecting about 300 million people worldwide [1]. The incidence of asthma is higher in high-income countries, whereas asthma-related mortality is predictably higher in lower-income countries [2]. Albeit the global prevalence of asthma is about 4–5%, this figure can vary as much as 21-fold across different countries [2]. The well-recognized pathophysiology of asthma consists of recurrent lung epithelial inflammation, bronchial smooth muscle hyperreactivity, chronic lung tissue remodeling, and excessive mucus production, leading to reversible airflow restriction [3]. The vast majority of patients with asthma also present with concomitant rhinitis, which can further amplify the risk for developing asthma with heightened bronchial hyperresponsiveness and reactivity to a variety of stimuli [4]. The current management of asthma primarily focuses on alleviating disease severity and choosing the appropriate medical therapy to control symptoms and reduce the risk of exacerbations; however, some patients still experience acute exacerbation of symptoms and a loss of disease control [5, 6], highlighting an urgent need to fully elucidate the underlying pathogenesis and develop more efficacious treatment regimens to better tackle asthma [7].

Recent data demonstrated that Yes-associated protein (YAP), a downstream target of the Hippo pathway, is implicated in the generation and maintenance of cancer-associated fibroblasts and vascular smooth muscle cell differentiation [8, 9]. YAP holds significant potential as an anticancer immunotherapeutic target owing to the fact that YAP deficiency induces dysfunctional Tregs unable to suppress anti-tumor immunity or promote tumor growth [10]. Silencing YAP has been shown to facilitate airway smooth muscle cell proliferation, migration, and contraction induced by sphingosine-1-phosphate in asthma [11]. In addition, YAP can bind to hypoxia-inducible factor 1α (HIF-1α) and maintain its protein stability, thus promoting hepatocellular carcinoma cell glycolysis under hypoxic stress [12]. During the induction phase of asthma, treatment with HIF-1α inhibitor is shown to decrease eosinophilia in bronchoalveolar lavage samples, lung parenchyma, and total lung inflammation [13]. Moreover, HIF-1α has also been shown to promote the expression of microRNA-182 (miR-182) [14]. This is notable as miR-182 is also documented as upregulated during T-helper 17 (Th17) cell differentiation [15]. Accruing research has revealed a significant correlation of activated Th17 cells with the progression of asthma [16–18]. Moreover, early growth response 2 (EGR2), known as a transcription factor negatively regulating T-cell activation [19], has been reported to reduce Th17 cell differentiation [20]. In another instance, a reduction of high-density lipoprotein cholesterol (HDL-C) has been linked to increased numbers of Th17 cells [21]. In a related finding, decreased HDL-C levels have been found in children with asthma [22]. Therefore, we sought to determine if YAP and HIF-1α function through a pathway that involves low HDL-C-induced increased Th17 cells in the pathogenesis of pediatric asthma. These results could potentially reveal a hitherto undocumented link between HIF-1α, miR-182, EGR2, and Th17 cells in context of asthma and therefore, we investigated if miR-182 and EGR2 were involved in asthma pathogenesis.

## Results

### YAP, HIF-1α and miR-182 are upregulated and HDL-C levels are decreased in asthma

With an aim to identify the putative genes implicated in the development of asthma, a differential gene expression analysis was performed on the asthma-related GSE27876 dataset retrieved from the Gene Expression Omnibus database (https://www.ncbi.nlm.nih.gov/gds) using the R package Limma (http://www.bioconductor.org/packages/release/bioc/html/limma.html). The GSE27876 dataset (comparing peripheral blood cells from mild and severe asthma that were selected from patients classified into the asthma treatment step 4, in accordance with the criteria described in the Global Initiative for Asthma) was identified. It comprised of 5 normal samples and 10 asthma samples where peripheral blood was the sample type. With the selected cutoff criteria (|logFoldChange|> 1.5, and *p* < 0.05), a total of 331 differentially expressed genes (DEGs) were identified (Additional file 1: Fig. S1A, Additional file 9: Table S1). In addition, 318 human transcription factors were identified from the Cistrome database (http://cistrome.org/). A Venn diagram analysis of the 331 DEGs and 318 human transcription factors revealed 5 transcription factors were found at the intersection, namely H2AFX, NOTCH1, RUNX1T1, USF1, and YAP1 (Additional file 1: Fig. S1B, Additional file 10: Table S2). Subsequently, protein–protein interactions (PPI) between these aforementioned 5 transcription factors and their related genes were identified using the GeneMANIA web-based tool (http://genemania.org/). As illustrated in Additional file 1: Fig. S1C and Table 1, the 2 most robustly related transcription factors were identified as NOTCH1 and YAP1. YAP1 has been widely correlated with asthma [23, 24], and also known to influence disease progression by mediating HIF-1α in liver cancer and pancreatic cancer [12, 25]. As demonstrated previously, NOTCH1 is involved in the promotion of the GATA3-mediated Th2 response (immunity), which makes NOTCH1 an unlikely candidate responsible for the non-Th2 inflammatory pattern in asthma [26]. As a result, YAP1 (YAP) was selected as the upstream gene for investigation in the current study. In addition, using the Multi Experiment Matrix (MEM) web based tool (https://biit.cs.ut.ee/mem/index.cgi) it was predicted that YAP1 was significantly co-expressed with HIF1A (Additional file 1: Fig. S1D). Using the hTFtarget tool (http://bioinfo.life.hust.edu.cn/hTFtarget#!/) YAP1 was found to target the HIF1A gene (Additional file 1: Fig. S1E). Based on these aforementioned in silico analyses and previous experimental data, we hypothesized that YAP1 (YAP) may affect the development of asthma through regulation of HIF1A (HIF-1α), and consequently designed the current study in order to validate this assumption.

**Table 1:** Sequences for mimic and inhibitor

Target gene	Sequence (5′ – 3′)
sh-YAP-1	AGTGCAGCAGAATATGATG
sh-YAP-2	GAGATGGATACAGGTGATA
sh-HIF-α-1	F: GATCCCCATCCAGAAGTCACTGGAACTTTCAAGAGAAGTTCCAGTGACTCTGGATTTTTGGAAA R: TCGAAAAGGTTTTTTTAGGTCTCAGTGACCTTGAAGAGAACTTTCAAGGTCACTGAGACCTACCC
sh-HIF-1α-2	F: AATTGATGGAACATGATGGTTCACTTCAAGAGAGTGAACCATCATGTTCCATTTTTT R: CTAGAAAAAAATGGAACATGATGGTTCACTCTCTTGAAGTGAACCATCATGTTCCATC
miR-182 inhibitor	TTCTACCATTGCCAA′
sh-EGR2-1	F: GATCCATGCGTAACTTCAGTCGTAAGAGAACTTTACGACTGAAGTTACGCATTTTTTTCTCGAGG R: AATTCCTCGAGAAAAAAATGCGTAACTTCAGTCGTAAAGTTCTCTTACGACTGAAGTTACGCATG
miR-182 mimic	F: UUUGGCAAUGGUAGAACUCACACU R: UGUGAGUUCUACCAUUGCCAAAUU

*YAP* Yes-associated protein, *HIF-1α* hypoxia-inducible factor 1α, *miR* microRNA, *EGR2* early growth response 2, *F* forward, *R* reverse

**Fig. 1:**
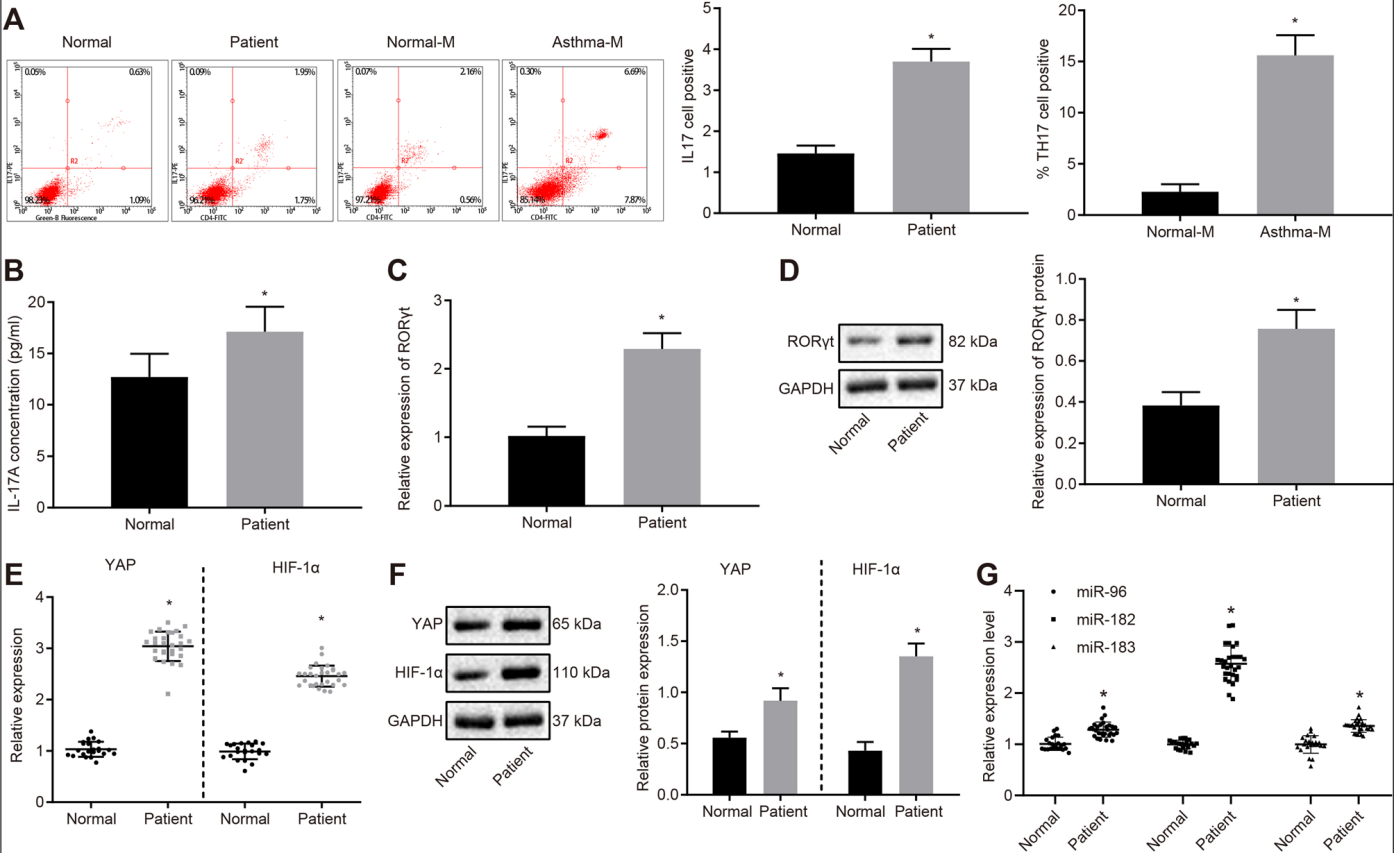
Increased YAP, HIF-1α and miR-182 and decreased HDL-C level identified in asthma. **a** Th17 cell proportion in human PBMCs and mouse spleen cells detected by flow cytometry; **b** the serum level of IL-17A in asthma patients measured by ELISA; **c** RORγt mRNA expression in human PBMCs determined by RT-qPCR; **d** Western blot analysis of RORγt protein in human PBMCs; **e** mRNA expression of YAP and HIF-1α in human PBMCs determined by RT-qPCR; **f** Western blot analysis of YAP and HIF-1α proteins in human PBMCs; **g** miR-183/96/182 expression in human PBMCs determined by RT-qPCR. Comparisons between two groups were conducted by unpaired *t* test; * *p* < 0.05, compared with the normal individuals (normal); n = 22 in normal individuals; n = 29 in asthma patients. Each experiment was repeated 3 times independently

The results of hematoxylin–eosin staining illustrated that the ovalbumin (OVA)-induced asthmatic mice presented with airway remodeling (*p* < 0.05; Additional file 2: Fig. S2A) and increased thickening of the airway smooth muscles, airway wall, and airway epithelium mucosa relative to the sham-operated mice (*p* < 0.05; Additional file 2: Fig. S2B). These findings verified the successful establishment of OVA-induced murine asthma models. Next, the proportion of Th17 cells was assessed in human peripheral blood mononuclear cells (PBMCs) and mouse spleen cells using flow cytometry, which revealed an increased proportion of Th17 cells in both patients and mice with asthma (*p* < 0.05; Fig. 1a). Furthermore, enzyme linked immunosorbent assay (ELISA) displayed that the serum levels of interleukin-17A (IL-17A) were up-regulated in patients with asthma (*p* < 0.05; Fig. 1b). In addition, reverse transcription quantitative polymerase reaction (RT-qPCR) and Western blot analysis determined that retineic-acid-receptor-related orphan nuclear receptor gamma (RORγt) mRNA and protein expression were elevated in PBMCs of patients with asthma (*p* < 0.05; Fig. 1c, d).

Using RT-qPCR and Western blot analysis YAP and HIF-1α expression levels were found significantly higher in patients with asthma (*p* < 0.05; Fig. 1e, f). As a significant association between asthma and the serum HDL-C has been described [22], HDL-C was employed as a marker to assess asthma disease severity in the current study and as depicted in Fig. 1g, serum HDL-C levels were significantly elevated in patients with asthma (*p* < 0.05). Evidence further suggests that HIF-1α can promote the expression of miR-182 [14], whereas the miR-183/96/182 cluster is known to be significantly upregulated in Th17 differentiation [15]. Therefore, we speculated that the HIF-1α signaling pathway might promote Th17 differentiation by regulating miR-182. Therefore, RT-qPCR quantification of miR-96/182/183 expression levels in human PBMCs was performed, showing an upward trend in the miR-96/182/183 expression in patients with asthma, with a particularly pronounced increase in miR-182 expression (*p* < 0.05; Fig. 1h). Largely in agreement with the results observed in human PBMCs, in mice with asthma, serum IL-17A content was significantly increased (Additional file 3: Fig. S3A, *p* < 0.05), RORγt was upregulated in spleen cells (Additional file 3: Fig. S3B, C, *p* < 0.05) accompanied by higher levels of YAP/HIF-1α (Additional file 3: Fig. S3D, E, *p* < 0.05), and lower HDL-C level (Additional file 3: Fig. S3F, *p* < 0.05) along with upregulation of miR-96/182/183 (*p* < 0.05), among which the increase in miR-182 expression was pronounced (Additional file 3: Fig. S3G, *p* < 0.05). Collectively, these results suggested that miR-182, YAP and HIF-1α were upregulated, and serum HDL-C level was decreased in asthma.

### YAP and HIF-1α promote the differentiation of CD4^+^ T Cells into Th17 cells

**Fig. 2:**
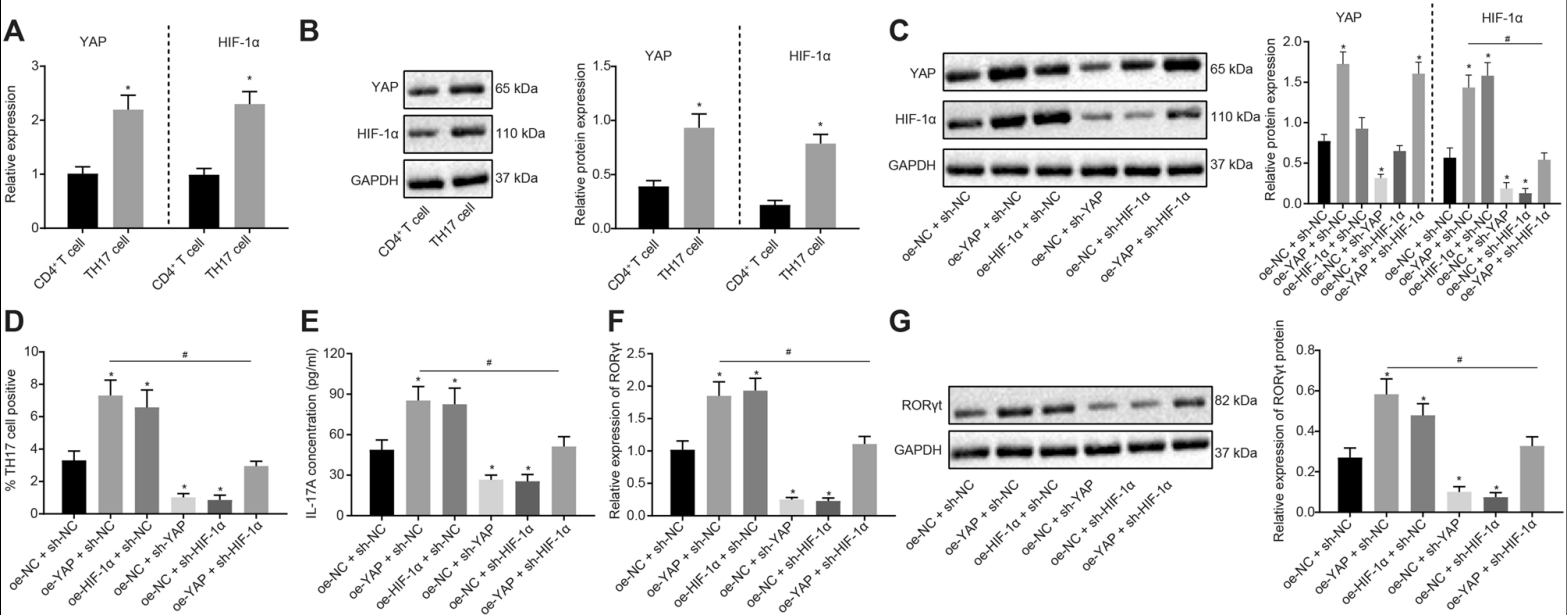
YAP and HIF-1α promote the differentiation of CD4^+^ T cells into Th17 cells. **a** mRNA expression of YAP and HIF-1α in Th17 cells determined by RT-qPCR; **b** Western blot analysis of YAP and HIF-1α proteins in Th17 cells; **c** Western blot analysis of YAP and HIF-1α proteins in Th17 cells following different treatments; **d** Th17 cell proportion after different treatments detected by flow cytometry; **e** IL-17A serum level in cell supernatant after different treatments by ELISA; **f** RORγt mRNA expression in cells after different treatments determined by RT-qPCR; **g** Western blot analysis of RORγt protein in cells after different treatments. Comparisons between two groups were conducted by unpaired *t* test, and those among multiple groups were conducted by one-way ANOVA with Tukey’s post hoc test; the experiments were repeated 3 times independently; * *p* < 0.05, compared with the CD4^+^ T cells, or cells treated with sh-NC, oe-NC, or sh-NC + oe-NC; # *p* < 0.05, compared with the cells treated with oe-YAP + sh-NC

In order to further verify whether YAP and HIF-1α were also upregulated in Th17 cells, we first induced the differentiation of CD4^+^ T cells into Th17 cells in vitro. Next, RT-qPCR and Western blot analysis were applied to determine the expression levels of YAP and HIF-1α in the differentiated TH17 cells. As illustrated in Fig. 2a, b, the mRNA and protein expression of YAP and HIF-1α were increased in Th17 cells compared to CD4^+^ T cells (*p* < 0.05).

Additionally, the infection efficiency of YAP/HIF-1α overexpression or knockdown in the TH17 cells was evaluated and confirmed by means of RT-qPCR and Western blot analysis (Additional file 4: Fig. S4A, B), where sh-YAP-1 and sh-HIF-1α-1 exhibited superior efficiency, and were thus used for further experimentation (*p* < 0.05). Western blot analysis demonstrated that the protein expression of HIF-1α was increased in cells with YAP overexpression, but decreased in YAP knockdown cells (*p* < 0.05; Additional file 4: Fig. S4C).

To further investigate whether YAP/HIF-1α promoted the differentiation of CD4^+^ T cells into Th17 cells, Western blot analysis was used to quantify protein expression levels of YAP and HIF-1α in Th17 cells subjected to different treatments. As shown in Fig. 2c, cells treated with overexpressed YAP or with a combination of YAP overexpression and HIF-1α silencing exhibited significantly increased YAP protein expression (*p* < 0.05). However, no significant changes were noted in the YAP protein expression levels between HIF-1α overexpressing cells and HIF-1α silenced cells (*p* > 0.05). When YAP or HIF-1α were overexpressed, HIF-1α expression was increased in Th17 cells (*p* < 0.05). The opposite trends were observed in Th17 cells upon YAP knockdown or/and HIF-1α knockdown treatment (*p* < 0.05).

Thereafter, the Th17 cell proportion was measured using flow cytometry (Fig. 2d), showing that YAP or HIF-1α overexpression led to higher Th17 cell proportion, while YAP or HIF-1α silencing resulted in reduced Th17 cell proportion (*p* < 0.05). In addition, increase in Th17 cell proportion caused by YAP overexpression was inhibited by HIF-1α silencing (*p* < 0.05).

The ELISA assays (Fig. 2e), RT-qPCR and Western blot analysis (Fig. 2f, g) revealed that IL-17A levels and RORγt expression were both elevated as a result of YAP or HIF-1α overexpression, but reduced in the absence of YAP or HIF-1α (*p* < 0.05). Additionally, increased IL-17A levels and RORγt expression owing to YAP overexpression were found to be inhibited by HIF-1α silencing (*p* < 0.05).

These findings demonstrated that the differentiation of CD4^+^ T cells into Th17 cells was promoted by overexpression of YAP and HIF-1α.

### HIF-1α promotes the differentiation of CD4^+^ T Cells into Th17 cells by upregulating miR-182

**Fig. 3:**
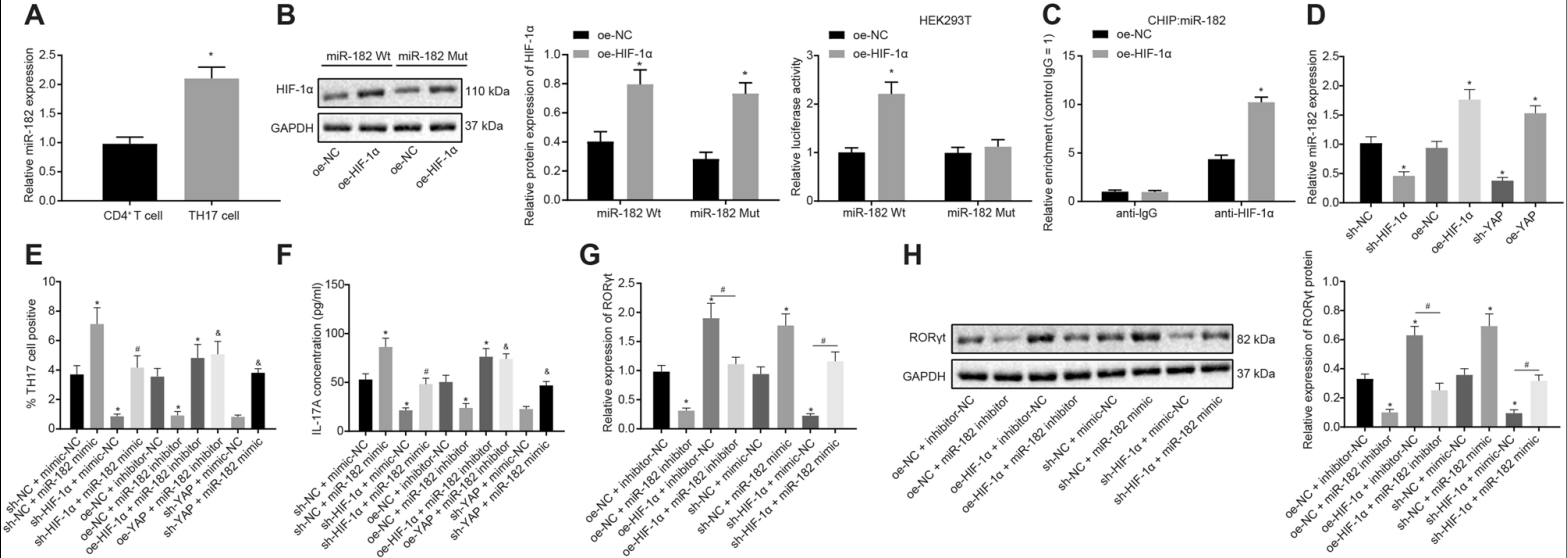
HIF-1α upregulates miR-182 and then promotes Th17 cell differentiation. **a** miR-182 expression in Th17 cells determined by RT-qPCR; **b** the HIF-1α protein expression by Western blot analysis and the effect of HIF-1α on the miR-182 promoter activity detected by dual luciferase reporter gene assay; **c** the binding of HIF-1α to miR-182 promoter detected by ChIP assay; **d** miR-182 expression after HIF-1α/YAP overexpression or knockdown determined by RT-qPCR; **e** Th17 cell proportion after different treatments detected by flow cytometry; **f** the level of IL-17A in cell supernatant after different treatments determined by ELISA; **g** RORγt mRNA expression in cells after different treatments determined by RT-qPCR; **h** Western blot analysis of RORγt protein in cells after different treatments. Comparisons between two groups were conducted by unpaired *t* test, and those between multiple groups were conducted by one-way ANOVA with Tukey’s post hoc test; the experiments were repeated 3 times independently; * *p* < 0.05, compared with the normal individuals (normal), normal mice (normal-M), CD4^+^ T cells, cells treated with sh-NC, oe-NC, oe-NC + inhibitor-NC, or sh-NC + mimic-NC; # *p* < 0.05, compared with the cells treated with oe-HIF-1α + inhibitor-NC or sh-HIF-1α + mimic-NC; & *p* < 0.05, compared with the cells treated with oe-YAP + inhibitor-NC or sh-YAP + mimic-NC. n = 22 in normal individuals; n = 29 in asthma patients; n = 12 in normal mice; n = 12 in asthma mice

In the following experiments, we aimed to uncover the mechanism of HIF-1α involvement in Th17 cell differentiation. RT-qPCR assay showed that miR-182 expression was upregulated in Th17 cells relative to that in CD4^+^ T cells (*p* < 0.05; Fig. 3a). In addition, Western blot analysis demonstrated that the protein expression of HIF-1α was elevated upon treatment with HIF-1α overexpression in HEK293T cells with miR-182-wild type (wt) or miR-182-mutant (mut) (*p* < 0.05). A dual luciferase reporter gene assay revealed that HIF-1α overexpression led to a significant increase in the luciferase activity of miR-182-wt in HEK293T cells (*p* < 0.05; Fig. 3b). Moreover, the chromatin immunoprecipitation (ChIP) assay illustrated that HIF-1α could bind to the miR-182 promoter (Fig. 3c), and compared to the immunoglobulin G (IgG) control, the enrichment of HIF-1α in the miR-182 promoter was notably increased, and was further promoted by HIF-1α overexpression (*p* < 0.05). In support, RT-qPCR assays demonstrated that the expression of miR-182 was increased in cells treated with oe-HIF-1α or oe-YAP, but was decreased in cells treated with sh-HIF-1α or sh-YAP (*p* < 0.05; Fig. 3d). These results indicated that HIF-1α promoted the expression of miR-182 in HEK293T cells.

Next, we examined whether YAP/HIF-1α promoted the differentiation of CD4^+^ T cells into Th17 cells. RT-qPCR assays showed reduced miR-182 expression in cells treated with oe-NC + miR-182 inhibitor, oe-HIF-1α + miR-182 inhibitor or oe-YAP + miR-182 inhibitor, and elevated miR-182 levels in cells treated with sh-NC + miR-182 mimic, sh-HIF-1α + miR-182 mimic or sh-YAP + miR-182 mimic (*p* < 0.05). RT-qPCR and Western blot analysis also revealed increased expression of HIF-1α in Th17 cells treated with oe-HIF-1α + miR-182 inhibitor, oe-HIF-1α + inhibitor-NC, oe-YAP + miR-182 inhibitor or oe-YAP + inhibitor-NC, with decreased levels in Th17 cells treated with sh-HIF-1α + miR-182 mimic, sh-NC + miR-182 mimic, sh-YAP + miR-182 mimic or sh-NC + miR-182 mimic (*p* < 0.05; Additional file 2 Fig. S2A, B).

The results of flow cytometry further displayed that Th17 cell proportion was reduced in response to treatment with oe-NC + miR-182 inhibitor, oe-HIF-1α + miR-182 inhibitor, oe-YAP + miR-182 inhibitor, sh-HIF-1α + mimic-NC or sh-YAP + mimic-NC, but opposite trends were noted upon treatment with sh-NC + miR-182 mimic, oe-HIF-1α + inhibitor-NC, oe-YAP + inhibitor-NC, sh-HIF-1α + miR-182 mimic or sh-YAP + miR-182 mimic (*p* < 0.05; Fig. 3e).

Further, ELISA assays demonstrated that IL-17A levels in the supernatant were decreased upon treatment with oe-NC + miR-182 inhibitor, which was reversed by sh-NC + miR-182 mimic, oe-HIF-1α + inhibitor-NC or oe-YAP + inhibitor-NC treatment. In addition, IL-17A levels were found to be diminished in the presence of oe-HIF-1α + miR-182 inhibitor or oe-YAP + miR-182 inhibitor. Silencing HIF-1α or YAP led to decreased IL-17A levels, while treatment with sh-HIF-1α + miR-182 mimic or sh-YAP + miR-182 mimic abrogated the trend (all *p* < 0.05; Fig. 3f).

RT-qPCR and Western blot analysis also illustrated that RORγt expression was diminished in cells upon miR-182 downregulation or HIF-1α downregulation, but elevated in cells treated with miR-182 upregulation or HIF-1α upregulation. Meanwhile, dual treatment with oe-HIF-1α and miR-182 inhibitor inhibited the RORγt expression, while treatment with both sh-HIF-1α and miR-182 mimic resulted in increased RORγt levels (*p* < 0.05; Fig. 3g, h).

These findings revealed that HIF-1α upregulated miR-182 expression and consequently promoted differentiation of CD4^+^ T cells into Th17 cells.

### EGR2 is downregulated in asthma

**Fig. 4:**
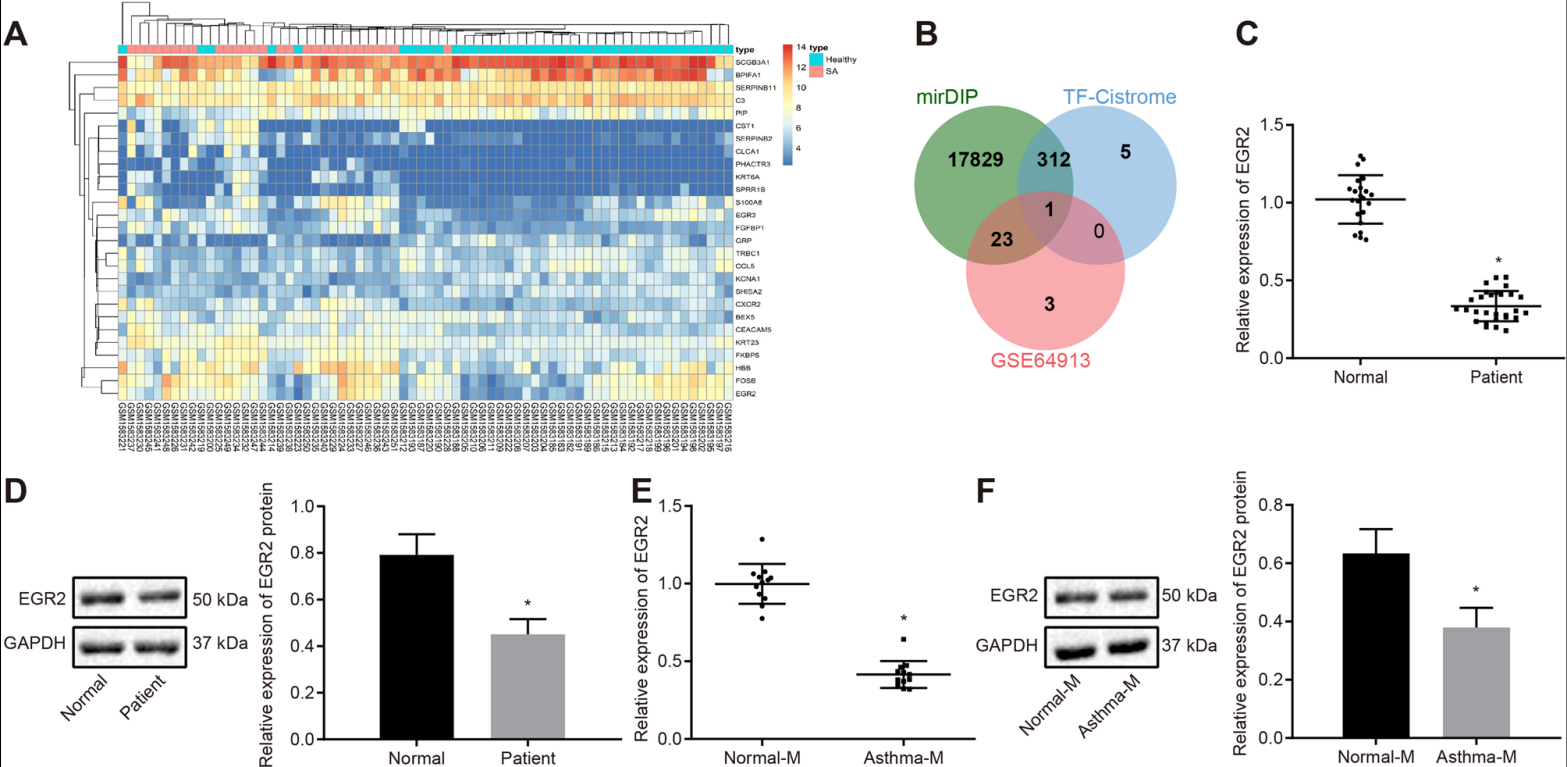
EGR2 expression is reduced in asthma. **a** the heatmap of DEGs related to asthma in respiratory epithelial cells, obtained from the GSE64913 dataset; the right upper histogram represents color gradation; **b** the Venn diagram showing the DEGs in respiratory epithelial cells from the GSE64913 dataset, downstream genes of miR-182 from the miRDIP database, and human transcription factors from the Cistrome database; **c** EGR2 mRNA expression in human PBMCs determined by RT-qPCR; **d** Western blot analysis of EGR protein in human PBMCs; **e** EGR2 mRNA expression in mouse spleen cells detected by RT-qPCR; **f** Western blot analysis of EGR2 protein in mouse spleen cells; Comparisons between two groups were conducted by unpaired *t* test. * *p* < 0.05, compared with the normal individuals (normal), normal mice (normal-M); n = 22 in normal individuals; n = 29 in asthma patients; n = 12 in normal mice; n = 12 in asthma mice. Each experiment was repeated 3 times independently

At the cutoff criteria (|logFoldChange|> 1, and *p* < 0.05), 27 DEGs were obtained from the GSE64913 dataset (epithelial cells from central airways and from peripheral airways), which included 42 normal samples and 28 asthma samples (Fig. 4a, Additional file 11: Table S3). Next, 18,165 downstream genes of miR-182 were identified from the mirDIP database (score class: medium; http://ophid.utoronto.ca/mirDIP/) and their human transcription factors in the Cistrome database were identified by means of a Venn diagram. EGR2 was found to be a key downstream transcription factor for miR-182 (Fig. 4b, Additional file 12: Table S4). EGR2 has also been reported to exert an inhibitory role on Th17 cell differentiation [21], and therefore, we evaluated its expression pattern in human PBMCs (Fig. 4c, d) and mouse spleen cells (Fig. 4e, f) using RT-qPCR and Western blot assays. The results demonstrated that EGR2 expression was downregulated in asthma (*p* < 0.05). As a result, we postulated that miR-182 might promote Th17 cell differentiation via inhibition of EGR2.

### miR-182 promotes the differentiation of CD4^+^ T Cells into Th17 Cells by inhibiting EGR2

**Fig. 5:**
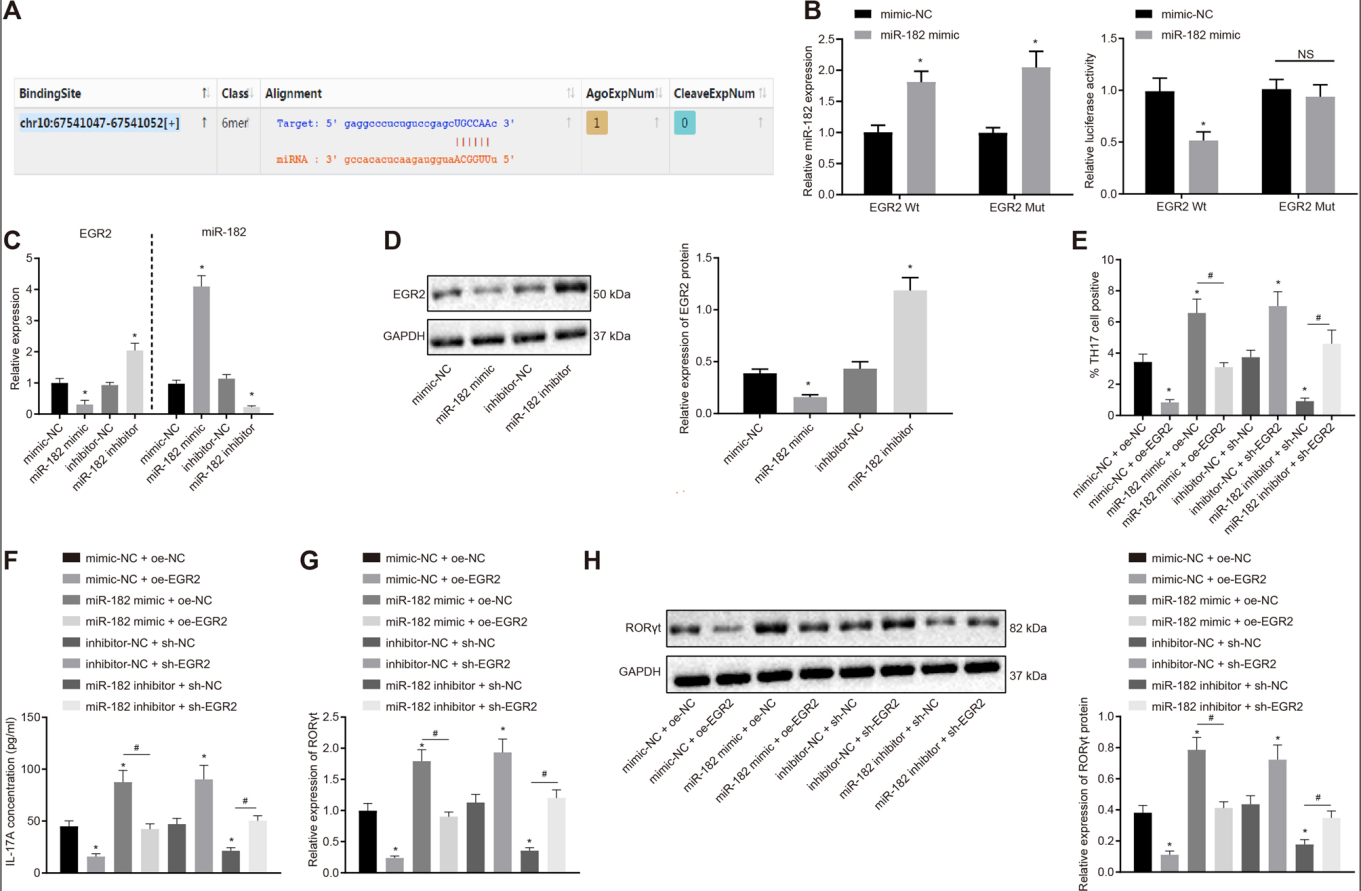
miR-182 inhibits EGR2 expression to promote Th17 cell differentiation. **a** the binding site between miR-182 and EGR2 3′UTR in mice predicted using starbase; **b** miR-182 expression determined by RT-qPCR and the binding of miR-182 to EGR2 confirmed by dual luciferase reporter gene assay; **c** miR-182 expression and EGR2 mRNA expression determined by RT-qPCR; **d** Western blot analysis of the EGR2 protein in cells after different treatments; **e** the Th17 cell proportion after different treatments determined by flow cytometry; **f** the level of IL-17A after different treatments by ELISA; **g** RORγt mRNA expression in cells after different treatments determined by RT-qPCR; **h** Western blot analysis of the RORγt protein in cells after different treatments. Comparisons between two groups were conducted by unpaired *t* test, and those between multiple groups were conducted by one-way ANOVA with Tukey’s post hoc test; the experiments were repeated 3 times independently; * *p* < 0.05, compared with cells treated with mimic-NC, inhibitor-NC, oe-NC + mimic-NC, or sh-NC + inhibitor-NC; # *p* < 0.05, compared with the cells treated with miR-182 mimic + oe-NC or miR-182 inhibitor + sh-NC

Next, we aimed to elucidate and verify the relationship between miR-182 and EGR2. The online resource (http://starbase.sysu.edu.cn/) was used to predict binding site presence between miR-182 and EGR2 3′untranslated region (3′UTR) (Fig. 5a). RT-qPCR analysis revealed that miR-182 expression was increased in HEK293T cells co-transfected with miR-182 mimic and EGR2-wt or EGR2-mut (*p* < 0.05). Thereafter, a dual luciferase reporter gene assay demonstrated that the luciferase activity of EGR2-wt was reduced (*p* < 0.05), while that of EGR2-mut showed no changes in HEK293T cells following miR-182 mimic transfection (*p* > 0.05; Fig. 5b). The results of RT-qPCR and Western blot analysis shown in Fig. 5c, d indicated a diminished expression of EGR2 in miR-182 mimic-treated cells and elevated expression of EGR2 in miR-182 inhibitor-treated cells (*p* < 0.05). These results reflected that miR-182 targeted EGR2 and inhibited its expression.

Going further, we examined whether miR-182 could induce Th17 cell differentiation via EGR2 inhibition. RT-qPCR and Western blot analysis showed that EGR2 expression was increased upon EGR2 overexpression but decreased following sh-EGR2-1 and sh-EGR2-2 treatment (*p* < 0.05; Additional file 6: Fig. S6A, B). Due to the higher silencing efficiency of EGR2-1 compared to EGR2-2, sh-EGR2-1 was chosen for subsequent experimentation.

Using RT-qPCR and Western blot analysis (Additional file 6: Fig. S6C, D), we showed that cells treated with oe-EGR2 or both miR-182 mimic and oe-EGR2 displayed elevated expression of EGR2, while those treated with sh-EGR2 exhibited reduced EGR2 (*p* < 0.05). Moreover, cells treated with miR-182 mimic showed increased miR-182 expression and decreased EGR2 expression (*p* < 0.05). However, cells treated with miR-182 mimic did not markedly differ in miR-182 expression from cells treated with both miR-182 mimic and oe-EGR2 (*p* > 0.05). In addition, cells treated with miR-182 inhibitor exhibited reduced miR-182 expression and elevated EGR2 expression (*p* < 0.05). However, as compared to the cells treated with miR-182 inhibitor, miR-182 expression was not significantly different from the cells co-treated with miR-182 inhibitor and sh-EGR2 (*p* > 0.05), while EGR2 was decreased (*p* < 0.05).

Flow cytometry was applied to assess the Th17 cell proportion in treated cells. As illustrated in Fig. 5e, EGR2 overexpression or miR-182 inhibitor treatment led to reduced Th17 cell proportion, while EGR2 knockdown or miR-182 mimic enhanced the Th17 cell proportion (*p* < 0.05). When compared with miR-182 mimic treatment alone, the combination treatment of miR-182 mimic and EGR2 overexpression resulted in a significantly decreased Th17 cell proportion (*p* < 0.05). At the same time, when compared with miR-182 inhibitor treatment alone, the combination treatment of miR-182 inhibitor and EGR2 silencing resulted in increased Th17 cell proportion (*p* < 0.05).

ELISA was performed to quantify IL-17A levels (*p* < 0.05; Fig. 5f), while RT-qPCR and Western blot analysis were conducted to determine the RORγt expression patterns (*p* < 0.05; Fig. 5g, h). IL-17A levels and RORγt expression were both decreased in cells with miR-182 downregulation or EGR2 upregulation, and elevated in cells with miR-182 upregulation or EGR2 downregulation (*p* < 0.05). When compared with miR-182 mimic treatment, the combination treatment of miR-182 mimic and EGR2 overexpression led to reduced IL-17A levels and RORγt expression (*p* < 0.05). When compared with miR-182 inhibitor treatment, the combination treatment of miR-182 inhibitor and EGR2 silencing exhibited elevated IL-17A levels and RORγt expression (*p* < 0.05).

Collectively, these findings suggested that miR-182 accelerated the differentiation of CD4^+^ T cells into Th17 cells by inhibiting the expression of EGR2.

### EGR2 inhibits the Th17 cell differentiation induced by YAP/HIF-1α/miR-182

**Fig. 6:**
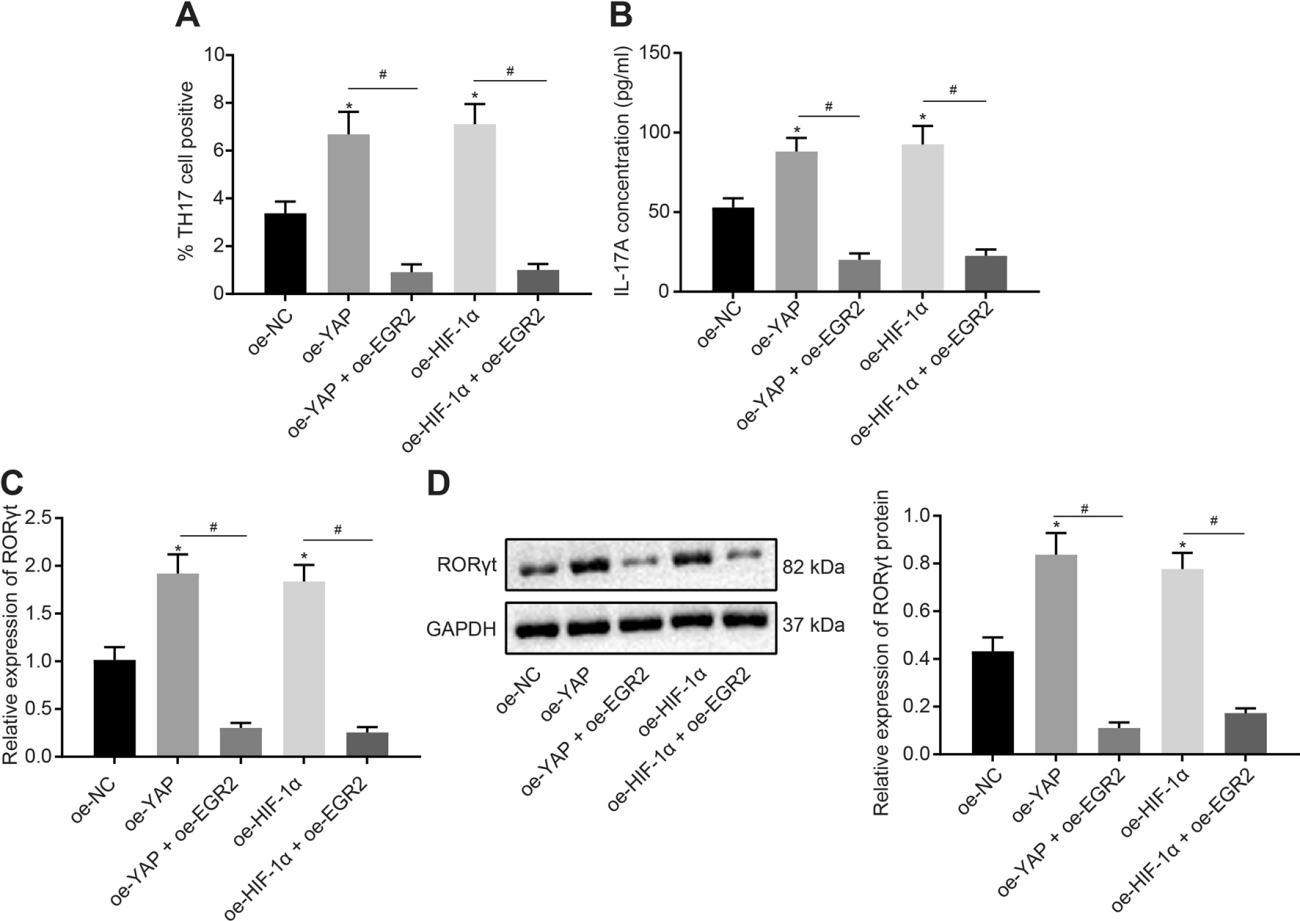
Overexpression of EGR2 inhibits Th17 cell differentiation induced by the YAP/HIF-1α/miR-182 signaling. **a** Th17 cell proportion after different treatments determined by flow cytometry; **b** the level of IL-17A in cell supernatant after different treatments measured by ELISA; **c** RORγt mRNA expression in cells after different treatments determined by RT-qPCR; **d** Western blot analysis of RORγt protein in cells after different treatments. Comparisons between multiple groups were conducted by one-way ANOVA with Tukey’s post hoc test; the experiments were repeated 3 times independently; * *p* < 0.05, compared with the cells treated with oe-NC; # *p* < 0.05, compared with the cells treated with oe-YAO or oe-HIF-1α

The aforementioned findings suggested that EGR2 overexpression could inhibit miR-182-induced Th17 cell differentiation. In this section of experiments, we investigated whether EGR2 inhibited the Th17 cell differentiation evoked by the YAP/HIF-1α axis. RT-qPCR and Western blot analysis (Additional file 7: Fig. S7A, B) displayed that the YAP expression was increased in CD4^+^ T cells upon YAP overexpression alone or both overexpression of YAP and EGR2 (*p* < 0.05). The HIF-1α expression was also increased upon HIF-1α overexpression alone or both overexpression of HIF-1α and EGR2 (*p* < 0.05). In addition, cells with both overexpression of YAP and EGR2 exhibited higher EGR2 expression relative to those with YAP overexpression alone, and the cells with both overexpression of HIF-1α and EGR2 also exhibited higher EGR2 expression compared to those with HIF-1α overexpression alone (*p* < 0.05). Flow cytometry results (Fig. 6a) depicted that YAP or HIF-1α overexpression promoted Th17 cell proportion, which was inhibited by EGR2 overexpression (*p* < 0.05).

Finally, Fig. 6b illustrates the results of IL-17A levels measured by ELISA and Fig. 6c, d display the RORγt expression patterns determined by RT-qPCR and Western blot analysis. IL-17A levels and RORγt expression were both elevated in cells with YAP or HIF-1α overexpression, while EGR2 overexpression led to a reversal of these trends (*p* < 0.05).

These data demonstrated that the differentiation of CD4^+^ T cells into Th17 cells induced by the YAP/HIF-1α/miR-182 axis was inhibited by overexpression of EGR2.

### Overexpression of EGR2 alleviates asthma and lipid metabolism dysfunction by inhibiting the YAP/HIF-1α/miR-182 axis in vivo

Next, we sought to verify the results in vivo. RT-qPCR was performed to determine the miR-182 expression patterns, and RT-qPCR and Western blot analysis were used to determine the protein expression patterns of YAP, HIF-1α, and EGR2 in mice (Additional file 8: Fig. S8A, B). Asthma mice presented with elevated YAP and HIF-1α expression, but reduced EGR2 expression (*p* < 0.05). Mice with YAP overexpression treatment or dual treatment with YAP over-expression and EGR2 overexpression exhibited increased expression of YAP (*p* < 0.05). In addition, the treatments with YAP overexpression alone, HIF-1α overexpression alone, both overexpression of YAP and EGR2, as well as both overexpression of HIF-1α and EGR2 resulted in increased expression of miR-182 and HIF-1α (*p* < 0.05). However, YAP overexpression, HIF-1α overexpression, or miR-182 overexpression resulted in decreased EGR2 expression, which could be rescued by EGR2 overexpression (*p* < 0.05). In addition, when compared with the mice treated with mimic-NC and oe-NC, the expression level of miR-182 was significantly higher in the mice treated with both EGR2 overexpression and miR-182 mimic (*p* < 0.05).

**Fig. 7:**
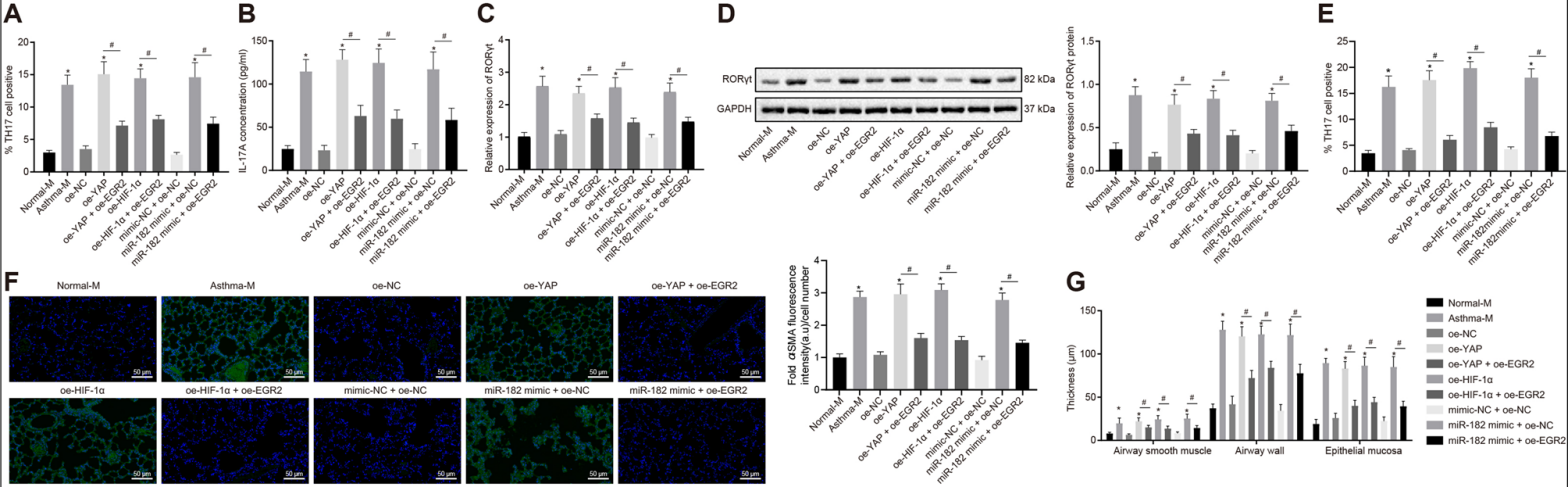
EGR2 overexpression alleviates asthma and lipid metabolism dysfunction induced by the YAP/HIF-1α/miR-182 axis in vivo. **a** the Th17 cell proportion in mouse spleen cells determined by flow cytometry; **b** the level of IL-17A in mouse serum measured by ELISA; **c** RORγt mRNA expression in mouse spleen cells determined by RT-qPCR; **d** Western blot analysis of RORγt protein in mouse spleen cells; **e** Th17 cell proportion in mouse lung tissues detected by flow cytometry. **f **Immunofluorescence staining showing the expression of α-SMA in the lung tissues of asthma mice

Thereafter, the Th17 cell proportion in mouse spleen was assessed using flow cytometry. The results are displayed in Fig. 7a, showing an increased Th17 cell proportion in the asthma mice and the mice treated with overexpressed YAP, overexpressed HIF-1α, or overexpressed miR-182, while inhibited by overexpressed EGR2 (*p* < 0.05). In addition, ELISA assessment of IL-17A levels (Fig. 7b) and RT-qPCR and Western blot to determine the RORγt expression patterns (Fig. 7c, d) showed IL-17A levels and RORγt expression were elevated in the asthma mice and the mice with overexpressed YAP, overexpressed HIF-1α, or overexpressed miR-182, which was inhibited by EGR2 overexpression (*p* < 0.05).

The Th17 cell proportion in mouse lung tissues evaluated using flow cytometry showed an increase in the asthma mice and the mice with overexpressed YAP, overexpressed HIF-1α, or overexpressed miR-182, which was, however, counteracted by the treatment with oe-YAP + oe-EGR2, oe-HIF-1α + oe-EGR2 or miR-182 mimic + oe-EGR2 (*p* < 0.05; Fig. 7e). Besides, as α-SMA immunofluorescence staining results show, the expression of α-SMA was elevated in the lung tissues of asthma mice, which was also increased in the asthma mice treated with overexpressed YAP, overexpressed HIF-1α, or overexpressed miR-182, whereas EGR2 overexpression significantly inhibited the expression of α-SMA (Fig. 7f). Furthermore, hematoxylin–eosin staining results demonstrated significant airway remodeling and increased thickening of the airway smooth muscles, airway wall, and airway epithelium mucosa in the asthma mice and mice with overexpressed YAP, overexpressed HIF-1α, or overexpressed miR-182, which was significantly inhibited by EGR2 overexpression (*p* < 0.05; Fig. 7g).

HDL-C level was found decreased in serum from patients with asthma (Additional file 5: Fig S5A). In addition, asthma mice treated with oe-YAP, oe-HIF-1α, or miR-182 mimic presented with reduced HDL-C levels, while increased HDL-C levels were found in response to EGR2 overexpression (*p* < 0.05; Additional file 5: Fig S5B).

These findings indicated that the overexpression of EGR2 alleviated the exacerbated asthma and lipid metabolism dysfunction evoked by YAP/HIF-1α/miR-182 signaling in vivo.

## Discussion

Asthma is a common disease affecting millions of people worldwide, characterized by upper airway inflammation and chronic nature [27]. In the current study, we aimed to investigate a potential molecular mechanism active in the development of asthma, and found that the YAP/HIF-1α factors could potentially augment Th17 cell differentiation, consequently exacerbating asthma and lipid metabolism dysfunction via miR-182-mediated EGR2 inhibition, thus providing a theoretical basis that could direct therapeutic strategies against asthma and lipid metabolic disorder.

One of the important findings of the current study is that the factors YAP1 and HIF-1α were both increased in pediatric asthma. A previous study corroborated that YAP was upregulated in the bronchial airway smooth muscles of chronic asthma mouse models [28]. YAP can also bind to HIF-1α in the nucleus and sustain HIF-1α protein stability in conditions of hypoxic stress in hepatocellular carcinoma cells [12]. Silencing YAP has been documented to markedly downregulate the protein expression of HIF-1α, while inhibition of the YAP/HIF-1α signaling aids in the prevention of angiogenesis of liver sinusoidal endothelial cells [29]. These previous findings in conjunction with our results support a positive relationship exists between YAP and HIF-1α. Other studies have illustrated that deficiency of HIF-1α can reduce eosinophil infiltration, goblet cell hyperplasia, and levels of cytokines IL-4, IL-5, and IL-13 in the lungs of OVA-induced asthma models [13], further highlighting the importance of the elevated levels of HIF-1α in asthma. Moreover, HIF-1α has been reported to facilitate the differentiation of Th17 cells [30], and may serve as an important signaling molecule for the induction of asthma by promoting Th17 cells [16, 17]. HIF-1α can also cause asthma by means of airway smooth muscle remodeling [31, 32], which is in line with our findings. Notably, YAP is known to function as an amplifier of the regulatory T cells Treg-reinforcing pathway, holding significant potential as an anticancer immunotherapeutic target [10]. In addition to that, loss of YAP in T cells is known to result in enhanced T-cell activation, differentiation, and function, which translates in vivo to an improved ability for T cells to infiltrate and repress the development of tumors [33]. These aforementioned findings and results indicate the promoting role of YAP/HIF-1α in enhancing differentiation of Th17 cells.

Lipid metabolism dysfunction is another potential problem faced by patients plagued by asthma. Accordingly, reductions in good cholesterol levels of HDL-C have been found in asthmatic children [22]. In addition to reduced HDL-C, abnormally high LDL-C and triglycerides are also a common occurrence in pediatric asthma patients [34]. More importantly, the reduction in HDL-C levels has been linked to an increased number of Th17 cells [21]. The significance of this phenomenon is reflected by the fact that Th17 cells are associated with the production of IL-17, a highly pro-inflammatory cytokine [35]. Th17 cells also produce other pro-inflammatory cytokines such as IL-6 and tumor necrosis factor-α, which play trivial roles in the inflammatory cascade stimulated in the state of asthma [36]. In particular, Th17 cells are understood to increase immune hyper-responsiveness in an enhanced inflammatory state during asthma [37]. Ni et al*.,* have demonstrated that loss of YAP results in dysfunctional Treg cells that fail to inhibit anti-tumor immunity or elicit tumor growth in mice [10]. Additionally, YAP deficiency in T cells enhances T-cell activation, differentiation, and function, as well as improving T-cell responses in cancer [33]. By contrast, the results obtained from the present study revealed that YAP could potentiate Th17 cell differentiation both in PBMCs and mice with asthma. This discrepancy may be due to the differences attributable to the laboratory environment, study subjects, and the detection methods used. However, a previous study found significant overexpression and activation of YAP-1 in PBMCs collected from a total of 152 hepatocellular carcinoma cases, and showed that YAP-1 shares a positive correlation to the percentage of Treg cells; specifically, YAP-1 overexpression in hepatocellular carcinoma T cells induces immunosuppression by promoting Treg cell differentiation [38]. This finding is consistent with the results from this work. It indicates that the role of YAP in Th17 generation could be bidirectional, relevant to both overexpression or deletion.

Another focus of the current study was miRNAs, small non-coding RNA molecules that can modulate gene expression posttranscriptionally by interacting with the 3′UTR of specific target mRNAs [39]. Herein, we identified that miR-182 could bind to the 3′UTR of EGR2 and inhibit its expression. Accumulating evidence has shown that EGR2 exerts an inhibitory role on Th17 cell differentiation by negatively regulating Batf [20]. A previous study had found upregulated expression of inflammatory transcription factors, such as RORγt and Bhlhe40, in EGR2/3 deficient T cells under tolerogenic conditions [40]. Furthermore, EGR2 has shown the potential to retard the development of chronic rhinosinusitis induced by miR-150-5p in dendritic cells [41]. On the other hand, previous studies have demonstrated that miR-182 may promote asthma by stimulating inflammation [42]. Our results also support the notion that miRNAs, like miR-182, miR-30 [43] and miR-221 [44], could be potential candidate targets for the development of new treatments of asthma [45]. In line with our findings, miR-182 was found significantly upregulated in Th17 differentiation [15]. Therefore, we reasoned that miR-182 led to Th17 cell differentiation in pediatric asthma by targeting EGR2 as miR-182 is found capable of elevating the HIF-1α expression, thus promoting breast cancer cell proliferation and invasion [46], and such evidence implied positive correlation between miR-182 and HIF-1α. HIF-1α-deficient mice are shown to exhibit elevated metabolic rate, hyperventilation, and improved glucose and lipid homeostasis [47]. Based on these results we validated the hypothesis that amplified EGR2 eliminated Th17 cell differentiation, asthma, and lipid metabolism dysfunction driven by YAP/HIF-1α/miR-182 signaling.

## Conclusions

**Fig. 8:**
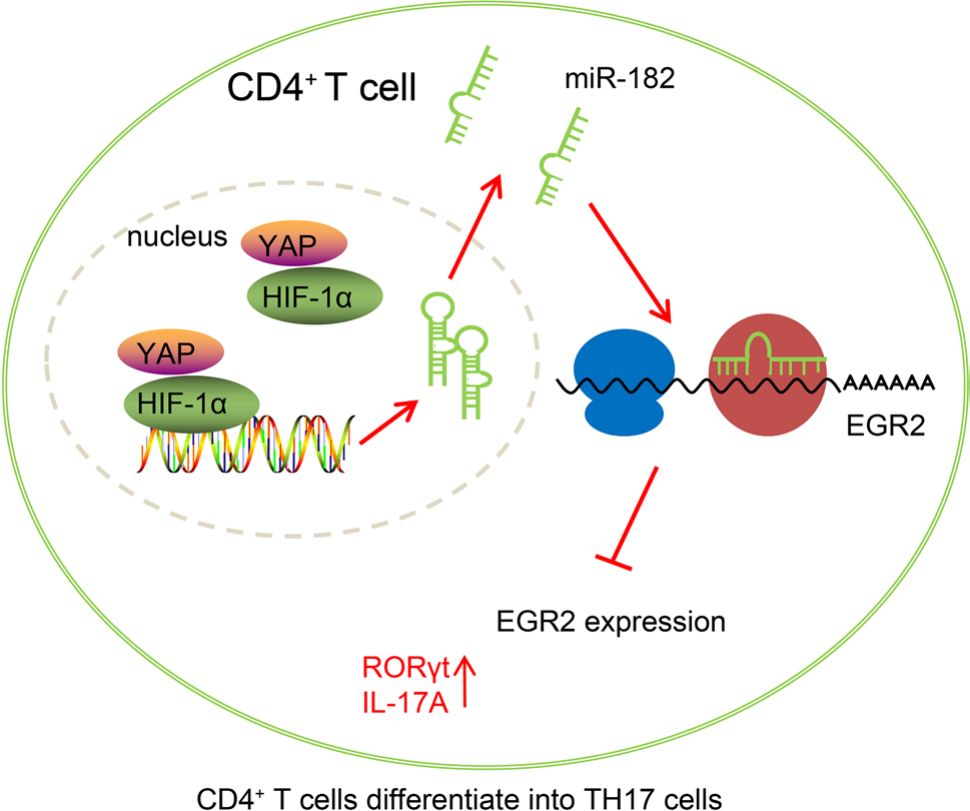
A schematic diagram illustrating the role of the YAP/HIF-1α/miR-182/EGR2 axis in asthma. The overexpression of YAP and HIF-1α promoted the expression of miR-182, thereby inhibiting EGR2 expression, increasing the expression of RORγt and IL-17A, and prompting the differentiation of CD4^+^T cells into Th17 cells, which ultimately aggravated asthma and lipid metabolism dysfunction

In conclusion, the findings in the current study demonstrated that YAP/HIF-1α enhanced Th17 cell differentiation, consequently exacerbating asthma and lipid metabolism dysfunction via miR-182-mediated EGR2 inhibition (Fig. 8). Thus, the YAP/HIF-1α/miR-182/EGR2 signaling may serve as a novel biomarker for asthma diagnosis and prognosis. However, there are a few notable limitations to our study. First, only OVA was used to stimulate asthma in the mice in this study. While human asthma can stem from different causes and exhibit variable forms and severity, animal models can be used to mimic one of more features of the human variation of the disease [48, 49] and therefore, in future, different animal models should be used to confirm the results uncovered in our study to prevent over-generalization and extrapolation to human situations. Also, in the mouse models with adenovirus-driven expression of YAP, HIF-1α, miR-182 and EGR2, it was hard to distinguish the effect of the expressed genes in different cell types, therefore, the epithelial cells from trachea to respiratory bronchioles, the alveolar ducks, and alveoli squamous type I alveolar cells, and type II alveolar cells should also be infected in future investigation. In general, further studies with different cell lines, different disease models, and larger cohorts are essential to validate the findings of the current study and validate the translational potential of the YAP/HIF-1α/miR-182/EGR2 signaling axis in asthma.

## Material and methods

### Ethics statement

The current study was approved by the Ethics Committee of The First Affiliated Hospital of Nanchang University (approval number: 201908029) and performed in strict accordance with the *Declaration of Helsinki*. Signed informed consent was obtained from all participants prior to their participation in the study. Animal experiments were strictly designed and performed in compliance with the Guide for the Care and Use of Laboratory animals published by the US National Institutes of Health, and extensive efforts were made to minimize the suffering of the animals used in the study.

### Study Subjects

A total of 29 pediatric patients with asthma (aged < 18 years old; 14 males & 15 females) and 22 healthy volunteers from the First Affiliated Hospital of Nanchang University from June 2018 to December 2018 were enrolled in the current study, and PBMCs were collected in order to extract RNA and protein content.

### OVA-Induced Murine Asthma Model

A total of 120 BALB/C mice (aged 6–8 weeks; weighing 16–20 g) were purchased from the Experimental Animal Center of Nanchang University, amongst which 12 mice were used as normal control, 96 mice were subjected to adenovirus infection, while the remaining 12 mice were used for asthma model establishment. The asthma models were constructed according to a previously reported method [50], with some additional adjustments. Firstly, the mice were subjected to intraperitoneal injections with 0.2 mL of OVA/aluminum hydroxide on days 0, 7, and 14. Thereafter, starting from day 21, the mice were exposed to 1% OVA inhalation (30 mL) for 30 min once per day, for a total of 7 days. At every time point prior to OVA inhalation, each mouse was intraperitoneally injected with 0.2 mL of normal saline. Normal control mice were subjected to matching procedures, with the exception of 30 min of OVA inhalation treatment. For the mice subjected to adenovirus infection, 1 week before administration of OVA and normal saline, the mice were intratracheally injected with phosphate buffer saline (PBS) or adenovirus harboring overexpression plasmids for YAP, EGR2, and HIF-1α, miR-182 mimic, or the negative control (NC) plasmids (20 μL, 5 × 10^10^ pfu/mL) (Fubio Biological Technology Co., Ltd., Shanghai, China) [51]. After model establishment, the mice were anaesthetized with intraperitoneal injections of 3% sodium pentobarbital (Sigma-Aldrich, St. Louis, MO, USA).

### Cell isolation and infection

HEK293T cells purchased from the American Type Culture Collection (Manassas, VA, USA) were cultured in high-glucose Dulbecco’s modified Eagle medium (DMEM) (10569044, Gibco, Langley, OK, USA) supplemented with 10% fetal bovine serum (FBS) (10099141, Gibco), 100 U/mL of penicillin, and 100 U/mL of streptomycin (15070063, Gibco).

Spleen specimens from normal BALB/c mice were collected, cut into pieces using surgical scissors and detached using Collagenase D for 30 min at room temperature, followed with treatment using 5 mM of ethylene diamine tetra-acetic acid for 5 min to harvest spleen cells. Next, naive CD4^+^ T cells were isolated from the harvested spleen cells with the help of CD4^+^ CD62L^+^ T cell isolation kits (130–106-643, Miltenyi Biotech, Germany), with a purity of 95%.

Subsequently, the obtained CD4^+^ T cells were seeded in a 6-well plate with Roswell Park Memorial Institute (RPMI)-1640 medium containing 10% FBS at a density of 5 × 10^5^ cells/well. Upon reaching 50–70% confluence, cells were treated with plasmids of miR-182 mimic, miR-182 inhibitor, overexpression and silencing adenoviruses carrying YAP, HIF-1α, and EGR2, as well as their corresponding NCs (mimic-NC, inhibitor-NC, oe-NC, and sh-NC) purchased from Sangon Biotech (Shanghai, China) for 24 h, followed by 1-week treatment with puromycin. After that, RT-qPCR and Western blot analysis were performed to measure the efficiency of cell infection. The sequences for mimic and inhibitor are listed in Table 2.

**Table 2:** Primer sequences for reverse transcription quantitative polymerase chain reaction

Primer	Sequence (5′–3′)
hYAP	F: TAGCCCTGCGTAGCCAGTTA R: TCATGCTTAGTCCACTGTCTGT
mYAP	F: CCCTCACCCTCCCTGAAATCTACAA
	R: AGCATTCCACAGGTCCAAGGCAGA
hHIF-1α mHIF-1α hmiR-182 mmiR-182 hEGR2 mEGR2 hRORγt mRORγt hGAPDH mGAPDH hU6 mU6	F: GCTGGCCCCAGCCGCTGGAG R: GAGTGCAGGGTCAGCACTAC F: AGCTTCTGTTATGAGGCTCACC R: TGACTTGATGTTCATCGTCCTC F: ACACTCCAGCTGGGTTTGGCAATGGTAGAACT R: TGGTGTCGTGGAGTCG F: TGCGGTTTGGCAATGGTAGAAC R: TGGTGTCGTGGAGTCG F: CTTTGACCAGATGAACGGAG R: CCCATGTAAGTGAAGGTCTG F: CTTCAGCCGAAGTGACCACC R: GCTCTTCCGTTCCTTCTGCC F: GCTGTGATCTTGCCCAGAACC R: TGCCCATCATCATTGCTGTTAATCC F: TGCAAGACTCATCGACAAGG R: AGGGGATTCAACATCAGTGC F: GGAGCGAGATCCCTCCAAAAT R: GGCTGTTGTCATACTTCTCAGG F: AGGTCGGTGTGAACGGATTTG R: TGTAGACCATGTAGTTGAGGTCA F: GTAATACGACTCACTATAGGGAGAAGAG R: CGCGCCTGCAGGTCGAC F: GCTTCGGCAGCACATATACTAAAAT R: CGCTTCACGAATTTGCGTGTCAT
hmiR-183	F: GAGGATCCCCGGGTACCAAGGGAGTGGGCAGGCTA R: ATAAGCTTGATATCGTCCCTGCACCCTTGGAAGCA
mmiR-183	F: CGCGCTAT GGCACTGGTAG
	R; GTGCAGGGTCC GAGGT
hmiR-96	F: TTC TTC TAG AGG AAG CCC TAA TCC
	R: TCC CTT CTA GAA AGA TCT ACT CCC C
mmiR-96	F: GCCC GCTTTGGCACTAGCACATT R: CAGTGCAGGGTCC GAGGT
hEGR2	F: CAGGTGTCCACTCCCAGGTCCAAG R: GGCAACTAGAAGGCACAGTCGAGG

*YAP* Yes-associated protein, *HIF-1α* hypoxia-inducible factor 1α, *miR* microRNA, *EGR2* early growth response 2, *F* forward, *R* reverse, *GAPDH* glyceraldehyde-3-phosphate dehydrogenase, *h* before gene name, Homo sapiens (human); m before gene name, Mus musculus (house mouse)

In order to differentiate the CD4^+^ T cells into Th17 cells, the infected CD4^+^ T cells were incubated in RPMI-1640 medium containing 10% FBS, 10 mM N-2-hydroxyethyl-piperazine-N'-2-ethanesulfonic acid, 100 U/mL of penicillin, 100 mg/mL of streptomycin, 50 mg/mL of gentamicin, 1 mM sodium pyruvate, 55 mM 2-ME, 1 mM non-essential amino acids, and 2 mM L-glutamine at 37 °C with 5% CO_2_ in air for a duration of 72 h. RPMI medium was prepared with the addition of anti-CD3 (10 μg/mL) (MAB4841), anti-CD28 (1 μg/mL) (MAB4832), transforming growth factor β (TGF-β) (2 ng/mL) (7666-MB), IL-6 (10 ng/mL) (406-ML), anti-IL-4 (10 μg/mL) (MAB404), and anti-interferon-γ (IFN-γ) (10 μg/mL) (485-MI), which were all purchased from R&D Systems (Minneapolis, MV, USA).

### Hematoxylin–eosin staining

Lung tissue sections were fixed with 4% paraformaldehyde at room temperature, and then subjected to hematoxylin–eosin staining (hematoxylin for 60 s and eosin for 3 min) for airway lesion observation. Each section was observed under an optical microscope (BX63, Tokyo, Japan) in a double-blinded manner, followed by measuring of the thickness of the airway smooth muscle (μm), airway wall (μm), and airway epithelium mucosa (μm).

### Preparation of single-cell suspension from lung tissue

Mice lungs were perfused with PBS and finely minced before treatment with digestion buffer containing Collagenase D (Roche) and MgCl_2_, and 0.15 mg/mL DNase I (Sigma) in DMEM (HyClone). Lung perfusion is a widely accepted method for the preparation of lung immune cells [52–54], including cells trapped in capillaries [55]. Lung perfusion was applied in our study to include both immune cells from lung parenchyma and also capillaries since tissue capillary-associated immune populations are pivotal to immune responses [56–58]. Lungs were digested for 20—25 min at 37 °C at 200 rpm and then passed through 100 mm cell strainer to prepare a single-cell suspension.

### Detection of Th17 cell proportion

Spleen tissues were isolated from C57BL/6 mice (age: 6—8 weeks), placed in a sterile culture dish, added with 5 mL autoMACS Running Buffer (Miltenyi Biotec) and rapidly ground as reported in a previous study [59]. A single-cell suspension was obtained by filtering through a 40 μm nylon membrane. Peripheral blood monocytes were isolated and collected from healthy humans. CD4^+^ CD25^−^ cell population was harvested through autoMACS Pro Cell Separator. Then, 1 × 10^5^ CD4^+^ CD25^−^ cells were added into a 96-well plate, and infected with adenovirus for 24 h. Each well was coated with CD3 antigen, washed with PBS 3 times and supplemented with 100 μL iTh17 mixture (1.5 μg/mL anti-CD28, 20 ng/mL IL-6 and 5 ng/mL TGF-β). The control mixture was activated and added as NC. After 72 h, Th17 cell differentiation was detected using a CytoFLEX flow cytometer (Beckman, Brea, CA, USA).

### RNA isolation and analysis

For measuring miRNA and mRNA expression, the total RNA was extracted from tissues and cells using the TRIzol reagent (6096020, Thermo Fisher Scientific, Waltham, MA, USA). After that, the obtained RNA was reverse transcribed into complementary DNA (cDNA) using TaqMan™ MicroRNA Reverse Transcription Kit (4366596, Thermo Fisher Scientific) for miR-182, and High-Capacity cDNA Reverse Transcription Kit (4368813, Thermo Fisher Scientific) for mRNAs. Then, RT-qPCR was performed using a RT-qPCR kit (11732020, Thermo Fisher Scientific) on a Real-Time PCR system (CFX96, Bio-Rad, Hercules, CA, USA). Finally, the expression levels of miRNA and mRNA were calculated using the 2^−ΔΔCt^ method, the internal reference set as U6 for miR-182 and glyceraldehyde-3-phosphate dehydrogenase (GAPDH) for YAP, HIF-1α, and EGR2. The primer sequences were designed by Shanghai Sangon Biotech (Shanghai, China) and are presented in Table 3.

**Table 3:** The degree of genes in PPI by GeneMANIA web-tool

Gene	Degree
NOTCH1	36
YAP1	33
H2AFX	15
RUNX1T1	15
USF1	10

*PPI* protein protein interaction, *YAP* Yes-associated protein

### Protein isolation and analysis

For assessment of protein expression, the total protein content was extracted from tissues and cells with a protein extraction kit (78501, Thermo Fisher Scientific), followed by determination of the protein concentration using a bicinchoninic acid kit (23229, Thermo Fisher Scientific). The protein was then electroblotted onto polyvinylidene difluoride membranes (1620177, Bio-Rad), which was blocked with 5% skim milk or 5% bovine serum albumin (BSA). The membranes were subsequently probed with the following primary antibodies at 4 °C overnight: GAPDH (internal control, ab181602, dilution ratio of 1: 5000, Abcam, Cambridge, UK), YAP (ab205270, dilution ratio of 1: 1000, Abcam), HIF-1α (ab2185, dilution ratio of 1: 1000, Abcam), EGR2 (ab108399, dilution ratio of 1: 1000, Abcam), and RORγt (MAB6109, dilution ratio of 1: 1000, R&D Systems). The following day, the membranes were re-probed with horseradish peroxidase-labeled secondary goat anti-rabbit IgG (ab6721, dilution ratio of 1: 5000, Abcam) or rabbit anti-mouse IgG (ab6728, dilution ratio of 1: 5000, Abcam) for 1 h at room temperature. After that, the membranes were visualized using an enhanced chemiluminescence reagent (1,705,062, Bio-Rad) and analyzed using Image Quant LAS 4000C (GE Company, USA). The protein expression was quantified using ImageJ 1.48u software (National Institutes of Health) and expressed as the gray value of the protein to be tested to that of GAPDH.

### Dual luciferase reporter gene assay

The possible binding site between miR-182 and the 3′UTR of EGR2 was predicted using a web based tool (http://starbase.sysu.edu.cn/). Next, synthesized fragments of EGR2-wt (5′-gaggccctctgtccgagctgccaac-3′), EGR2-mut (5′-gaggccctctgtccgagcacggttc-3′), miR-182-wt, and miR-182-mut (Ribo, Guangzhou, Guangdong, China) were introduced into a pGL3 vector (E1751, PromegaCorp., Madison, WI, USA) using T4 DNA ligase (M0204S, New England Biolabs Inc., MA, USA) and restriction endonuclease. These luciferase reporter plasmids were co-transfected with oe-NC, oe-HIF-1α, mimic-NC, and miR-182 mimic into HEK293T cells, respectively, and cultured for a duration of 48 h. The luciferase activity was measured using a Dual-Luciferase® Reporter Assay System kit (E1910, Promega) on a GLomax 20/20 Luminometer fluorescence detector (E5311, Promega). The luminescent signal reflecting the activation of the target reporter gene was computed using the ratio of the firefly relative light units (RLU) to the Renilla RLU. All vectors were constructed by Shanghai Sangon Biotechnology Co. Ltd. (Shanghai, China).

### HDL-C determination

The HDL-C content was determined according to the manual of the HDL-C detection kit (ab65390, Abcam).

### ELISA

Naive CD4 + T cells were infected with adenoviruses and differentiated in Th17 cell differentiation medium for 72 h. The levels of IL-17A in Th17 cells of mice were measured using an IL-17A ELISA kit (M17AF0, R&D Systems) according to the manufacturer’s instructions. The serum levels of IL-17A in human were measured according to the instructions provided in the ELISA kit (D1700, R&D Systems).

### ChIP assay

The ChIP assay was conducted using a EZ-Magna ChIP kit (EMD Millipore, USA) according to standard instructions. The CD4^+^ CD25^−^ cell population was activated to produce Th17 cells by iTh17mixture, followed by flow cytometric sorting to screen positive Th17 cells. In brief, 1 × 10^6^ Th17 cells were fixed with 1% paraformaldehyde and cross-linked with glycine for 10 min to produce DNA–protein cross-linking. The cells were then subjected to sonication to shear the DNA into 200–300 bp fragments. The supernatant was then collected and incubated with protein-A coated magnetic beads, followed by the addition of IgG (ab172730, dilution ratio of 1: 100, Abcam) or antibody against HIF-1α (ab2185, dilution ratio of 1: 20, Abcam). The protein-DNA complexes immobilized by magnetic beads were washed and de-crosslinked. Finally, the miR-182 promoter region in the complexes was determined by RT-qPCR (miR-182 ChIP primer: Forward: 5′-GAGTGTCCAGGGTTCGTCTG-3′, Reverse: 5′-GGTACACTTCTTTGCCCCCA-3′).

### Immunofluorescence staining

After rewarming for 30 min, the frozen lung tissue Sects. (5–7 μ) of mice were fixed with 4% formaldehyde for 15 min at room temperature, and then treated with 0.2% Triton X-100 at 4℃ for 30 min. Next, the sections were blocked using 1% BSA at room temperature for 60 min, and subsequently incubated with mouse polyclonal anti-α-SMA primary antibody (a2547, dilution ratio of 1: 400, Sigma) in stable buffer containing 1% BSA at room temperature for 60 min. Then the tissue sections were incubated with goat anti mouse secondary antibody (A-11001, dilution ratio of 1: 1000, Thermo Fisher Scientific) at room temperature for 60 min. Finally, the sections were incubated with 4,6-diamino-2-phenylindole (DAPI) (D523, dilution ratio of 1: 1000, DojinChemical Co., Kumamoto, Japan) for 10 min in the dark. Immunofluorescence images were collected using a laser scanning confocal microscope (A1RMP, Nikon, Tokyo, Japan). The fluorescence intensity was quantified and analyzed by Image-J software.

### Statistical analysis

The Statistic Package for Social Science 21.0 statistical software (IBM Corp., Armonk, NY, USA) was used for statistical analyses. Measurement data were expressed as mean ± standard deviation. Comparisons between two groups were performed using unpaired *t-*test, and comparisons between multiple groups were performed using one-way analysis of variance (ANOVA) with Tukey’s post-hoc test. A value of *p* < 0.05 was considered to be statistically significant.

## Supplementary Information

**Additional file 1**: **Figure S1**. YAP1 and HIF1A may be implicated in asthma. A, the volcano plot of DEGs related to asthma in peripheral blood cells of asthma patients obtained from the GSE97049 dataset. The red points indicate significantly upregulated genes, and the green points indicate significantly downregulated genes; B, the Venn diagram of the DEGs in peripheral blood cells of asthma patients from the GSE97049 dataset and the human transcription factors obtained from the Cistrome database; C, the PPI network of the 5 intersecting transcription factors in panel B and the related genes; the larger circle at which genes are located reflects higher core degree of the gene and the smaller circle reflects lower core degree. D, the co-expression of YAP1 and HIF1A predicted by the MEM website (p = 3.76e-18); E, the target relationship between YAP1 and HIF1A predicted by the hTFtarget website.

**Additional file 2**: **Figure S2**. A mouse model of asthma was successfully developed. A, Hematoxylin-eosin staining of mouse lung tissues (400 ×); B, diagram depicting the thickness of airway smooth muscle, airway wall, and airway epithelium mucosa of mice.

**Additional file 3**: **Figure S3**. YAP/HIF-1α is upregulated while HDL-C is downregulated in mice with asthma. A, the serum level of IL-17A in asthma mice measured by ELISA; B, RORγt mRNA expression in mouse spleen cells determined by RT-qPCR; C, Western blot analysis of RORγt protein in mouse spleen cells; D, mRNA expression of YAP and HIF-1α in mouse spleen cells determined by RT-qPCR; E, Western blot analysis of YAP and HIF-1α proteins in mouse spleen cells. F, the serum level of HDL-C in asthma mice; G, miR-183/96/182 expression in mouse spleen cells determined by RT-qPCR. Comparisons between two groups were conducted using unpaired t test. * p < 0.05, compared with normal mice (normal-M). n = 12. Each experiment was repeated 3 times independently.

**Additional file 4**: **Figure S4**. Efficiency of overexpression or knockdown of YAP/HIF-1α in Th17 cells. A, the mRNA expression of YAP and HIF-1α in cells detected by RT-qPCR; B, Western blot analysis of YAP and HIF-1α proteins in cells; C, Western blot analysis of HIF-1α proteins in cells after overexpressing/silencing YAP.

**Additional file 5**: **Figure S5**. Expression of miR-182 and HIF-1α in Th17 cells. A, HDL-C level in human serum. B, HDL-C level in mouse serum. C, miR-182 expression and HIF-1α mRNA expression in cells after different treatments determined by RT-qPCR; D, Western blot analysis of HIF-1α protein in cells after different treatments.

**Additional file 6**: **Figure S6**. Efficiency of EGR2 overexpression or knockdown in Th17 cells. A, the mRNA expression of EGR2 in cells determined by RT-qPCR; B, Western blot analysis of EGR2 protein in cells; C, the mRNA expression of EGR2 in cells after different treatments determined by RT-qPCR; D, Western blot analysis of EGR2 protein in cells after different treatments.

**Additional file 7**: **Figure S7**. Expression of YAP, HIF-1α and EGR2 in naive CD4+T cells. A, the mRNA expression of YAP, HIF-1α and EGR2 in cells after different treatments determined by RT-qPCR; B, Western blot analysis of YAP, HIF-1α and EGR2 proteins in cells after different treatments.

**Additional file 8**: **Figure S8**. Expression of YAP, HIF-1α, miR-182 and EGR2 in mouse spleen cells. A, miR-182 expression and the mRNA expression of YAP, HIF-1α, and EGR2 in mouse spleen cells after different treatments determined by RT-qPCR; B, Western blot analysis of YAP, HIF-1α and EGR2 proteins in mouse spleen cells after different treatments.

**Additional file 9: Table S1.** Differentially expressed genes identified in GSE27876 dataset.

**Additional file 10: Table S2.** Human transcription factors retrieved from the Cistrome database.

**Additional file 11: Table S3.** Differentially expressed genes obtained from the GSE64913 dataset.

**Additional file 12: Table S4.** Downstream genes of miR-182 identified from the mirDIP database.

## Abbreviations

YAP: Yes-associated protein
miR: MicroRNA
EGR2: Early growth response 2
PBMCs: Peripheral blood mononuclear cells
miR-182: MicroRNA-182
Th17: T-helper 17
HDL-C: High-density lipoprotein cholesterol
PBS: Phosphate buffer saline
NC: Negative control
EDTA: Ethylene diamine tetra-acetic acid
RPMI: Roswell Park Memorial Institute
RT-qPCR: Reverse transcription quantitative polymerase chain reaction
NEAA: Non-essential amino acids
FITC: Fluorescein isothiocyanate
GAPDH: Glyceraldehyde-3-phosphate dehydrogenase
BSA: Bovine serum albumin
3′-UTR: 3′-Untranslated region
RLU: Relative light units
ChIP: Chromatin Immunoprecipitation
ANOVA: Analysis of variance
DEGs: Expressed genes
PPI: Protein–protein interaction
MEM: Multi Experiment Matrix
