# Supplementary figures and images for: The YAP/HIF-1α/miR-182/EGR2 axis is implicated in asthma severity through the control of Th17 cell differentiation
Source: Cell Biosci. 2021 May 12;11:84. doi: 10.1186/s13578-021-00560-1 (PMC8117288; doi:10.1186/s13578-021-00560-1)

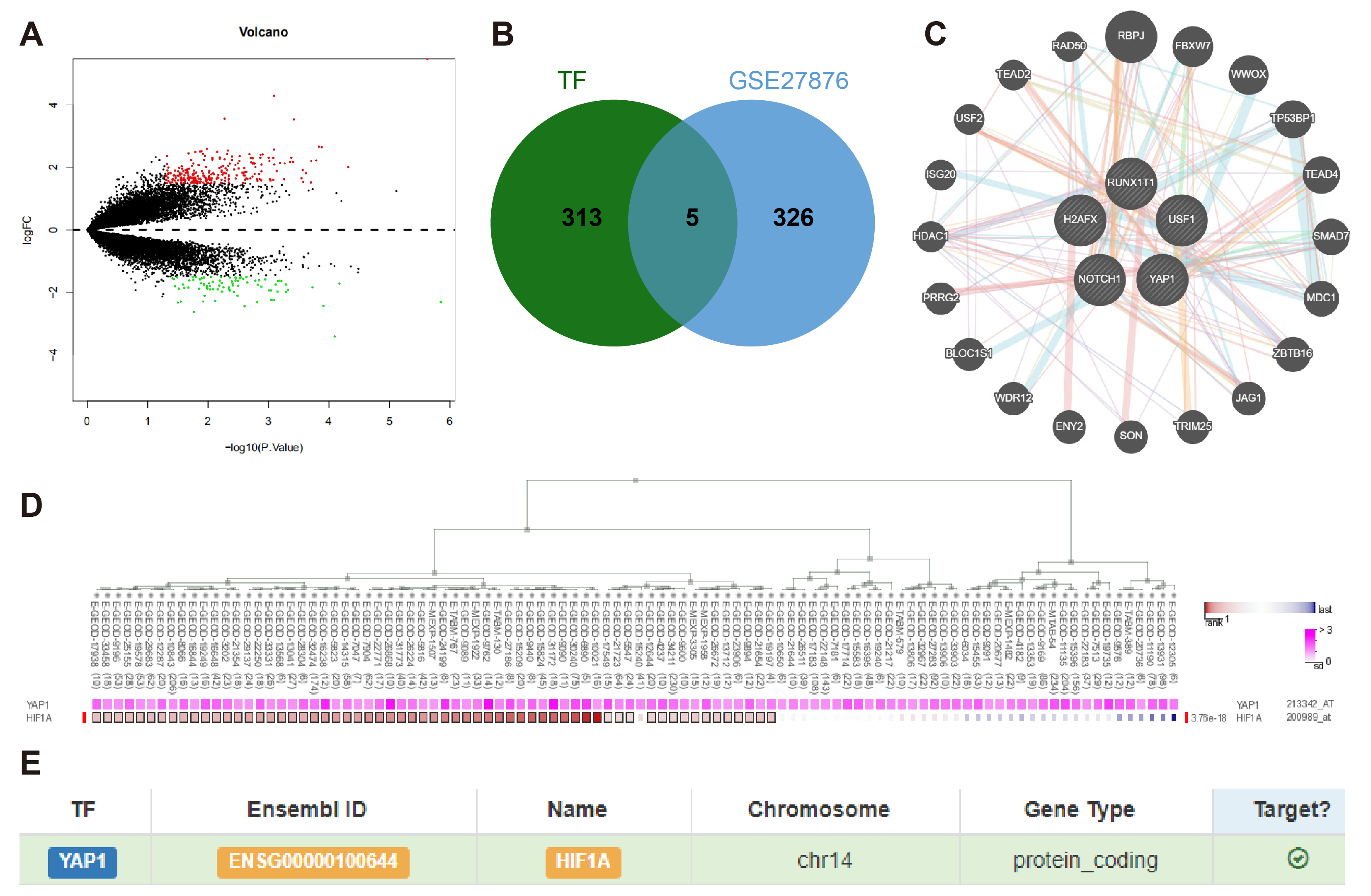

Supplement: Supplementary file 1 — Additional file 1: Figure S1. YAP1 and HIF1A may be implicated in asthma. A, the volcano plot of DEGs related to asthma in peripheral blood cells of asthma patients obtained from the GSE97049 dataset. The red points indicate significantly upregulated genes, and the green points indicate significantly downregulated genes; B, the Venn diagram of the DEGs in peripheral blood cells of asthma patients from the GSE97049 dataset and the human transcription factors obtained from the Cistrome database; C, the PPI network of the 5 intersecting transcription factors in panel B and the related genes; the larger circle at which genes are located reflects higher core degree of the gene and the smaller circle reflects lower core degree. D, the co-expression of YAP1 and HIF1A predicted by the MEM website (p = 3.76e-18); E, the target relationship between YAP1 and HIF1A predicted by the hTFtarget website. [file 13578_2021_560_MOESM1_ESM.jpg]

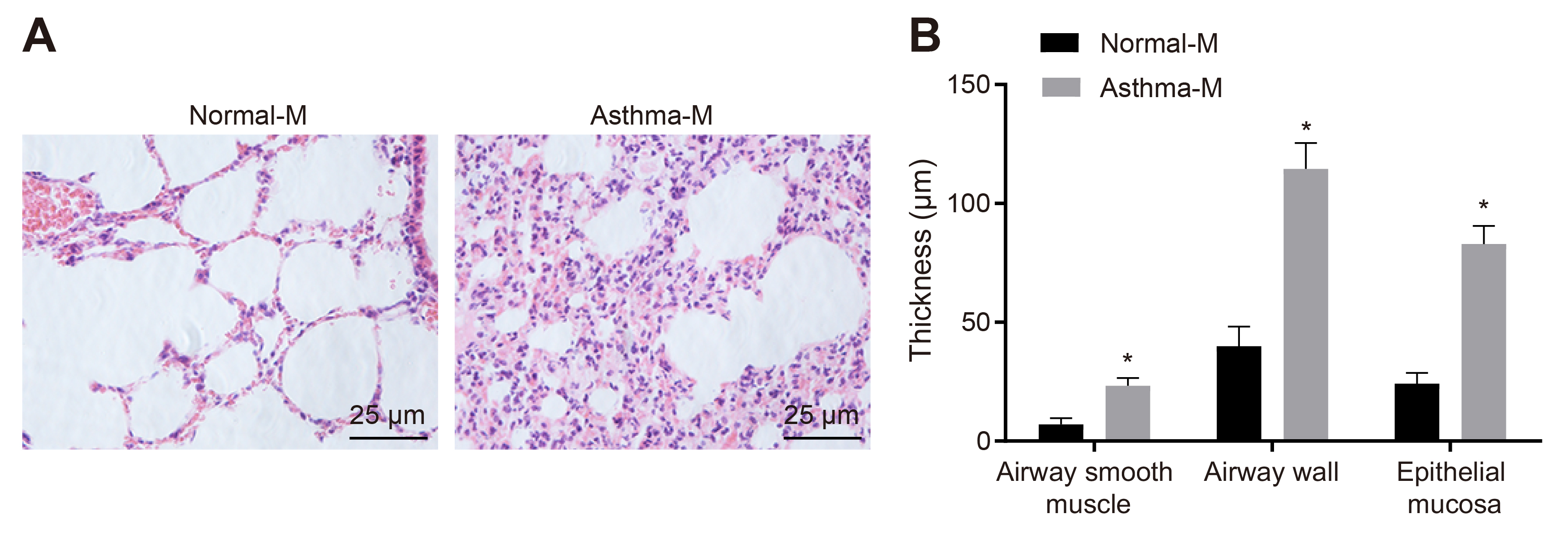

Supplement: Supplementary file 2 — Additional file 2: Figure S2. A mouse model of asthma was successfully developed. A, Hematoxylin-eosin staining of mouse lung tissues (400 ×); B, diagram depicting the thickness of airway smooth muscle, airway wall, and airway epithelium mucosa of mice. [file 13578_2021_560_MOESM2_ESM.jpg]

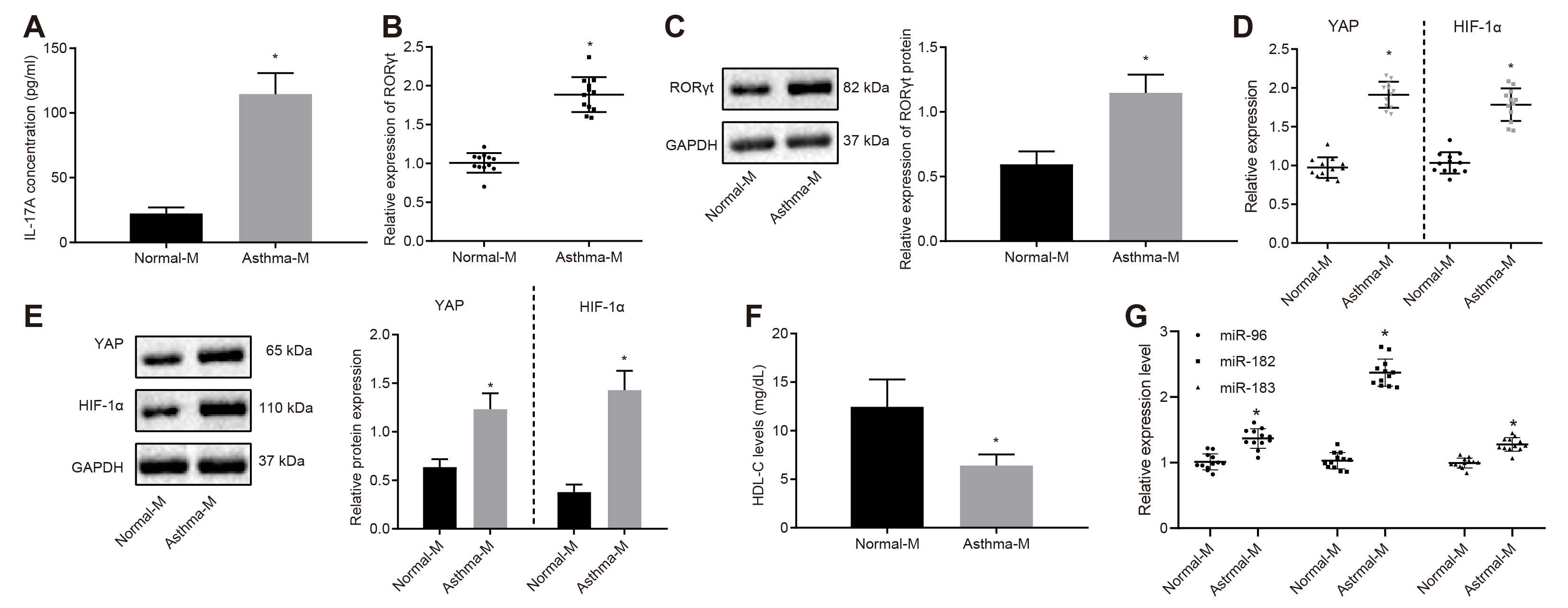

Supplement: Supplementary file 3 — Additional file 3: Figure S3. YAP/HIF-1α is upregulated while HDL-C is downregulated in mice with asthma. A, the serum level of IL-17A in asthma mice measured by ELISA; B, RORγt mRNA expression in mouse spleen cells determined by RT-qPCR; C, Western blot analysis of RORγt protein in mouse spleen cells; D, mRNA expression of YAP and HIF-1α in mouse spleen cells determined by RT-qPCR; E, Western blot analysis of YAP and HIF-1α proteins in mouse spleen cells. F, the serum level of HDL-C in asthma mice; G, miR-183/96/182 expression in mouse spleen cells determined by RT-qPCR. Comparisons between two groups were conducted using unpaired t test. * p < 0.05, compared with normal mice (normal-M). n = 12. Each experiment was repeated 3 times independently. [file 13578_2021_560_MOESM3_ESM.jpg]

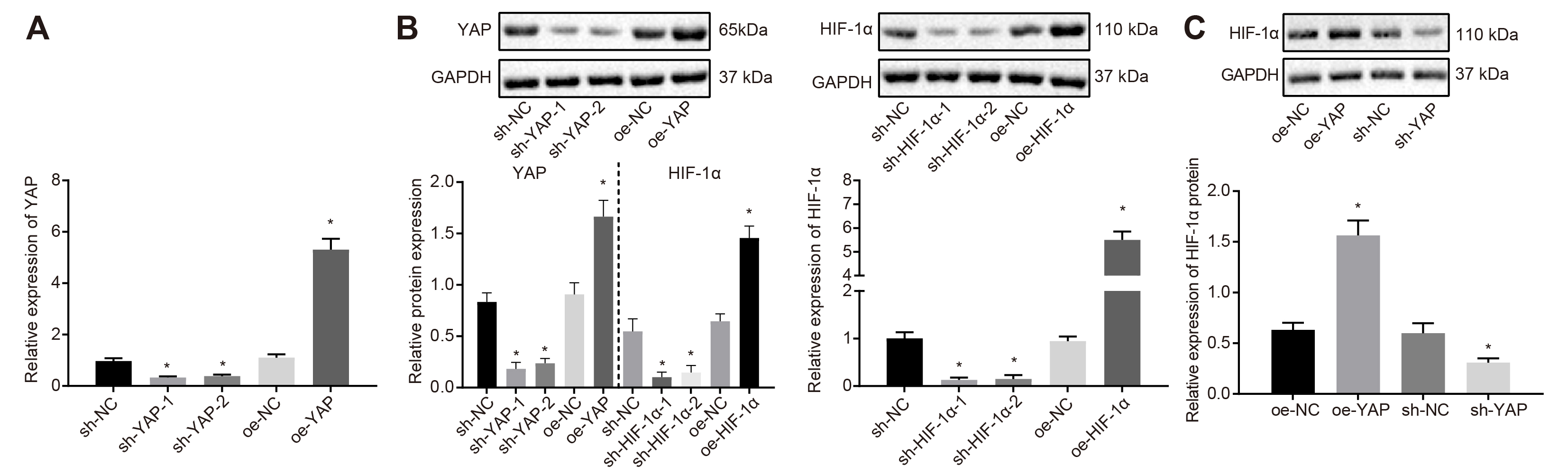

Supplement: Supplementary file 4 — Additional file 4: Figure S4. Efficiency of overexpression or knockdown of YAP/HIF-1α in Th17 cells. A, the mRNA expression of YAP and HIF-1α in cells detected by RT-qPCR; B, Western blot analysis of YAP and HIF-1α proteins in cells; C, Western blot analysis of HIF-1α proteins in cells after overexpressing/silencing YAP. [file 13578_2021_560_MOESM4_ESM.jpg]

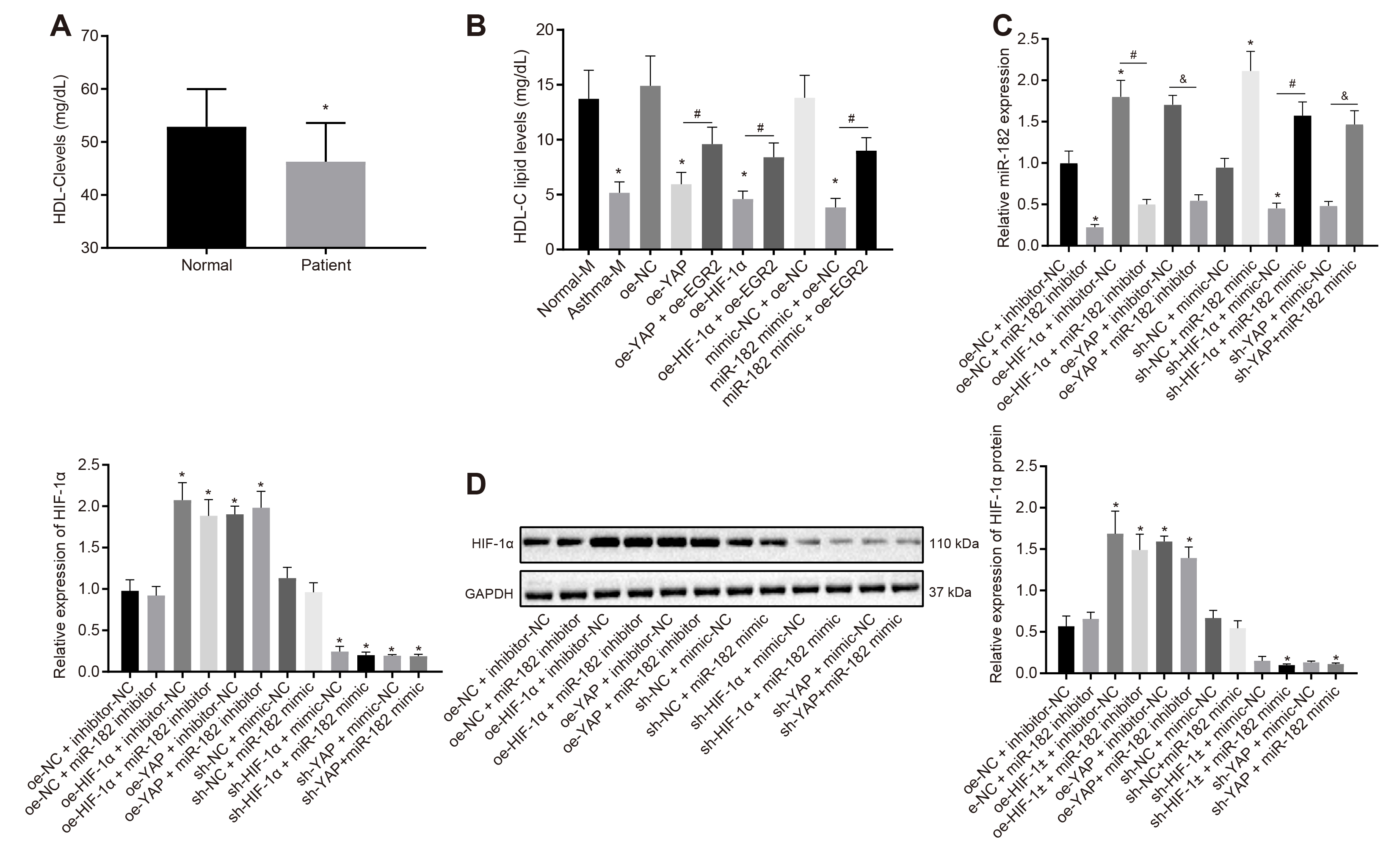

Supplement: Supplementary file 5 — Additional file 5: Figure S5. Expression of miR-182 and HIF-1α in Th17 cells. A, HDL-C level in human serum. B, HDL-C level in mouse serum. C, miR-182 expression and HIF-1α mRNA expression in cells after different treatments determined by RT-qPCR; D, Western blot analysis of HIF-1α protein in cells after different treatments. [file 13578_2021_560_MOESM5_ESM.jpg]

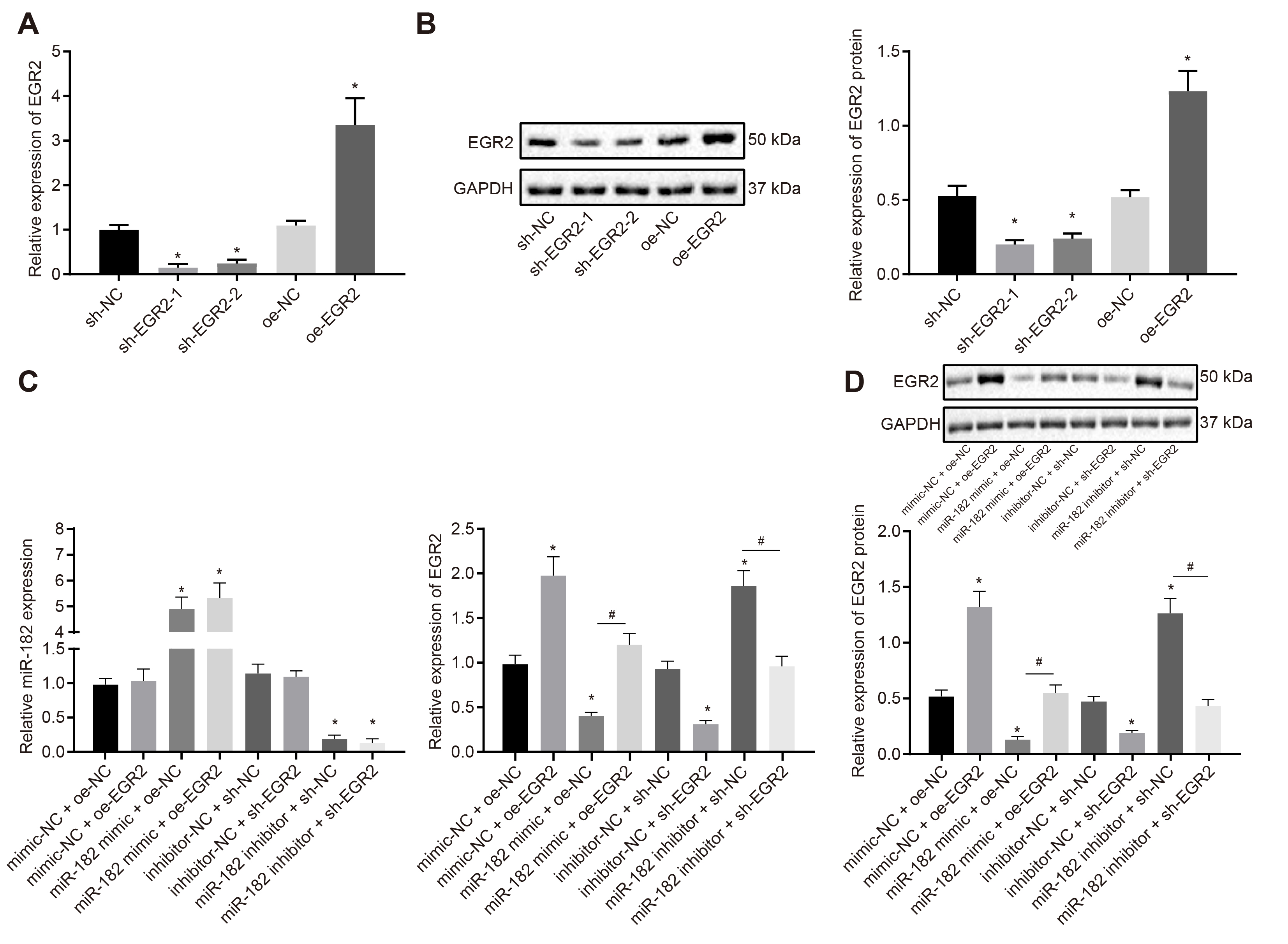

Supplement: Supplementary file 6 — Additional file 6: Figure S6. Efficiency of EGR2 overexpression or knockdown in Th17 cells. A, the mRNA expression of EGR2 in cells determined by RT-qPCR; B, Western blot analysis of EGR2 protein in cells; C, the mRNA expression of EGR2 in cells after different treatments determined by RT-qPCR; D, Western blot analysis of EGR2 protein in cells after different treatments. [file 13578_2021_560_MOESM6_ESM.jpg]

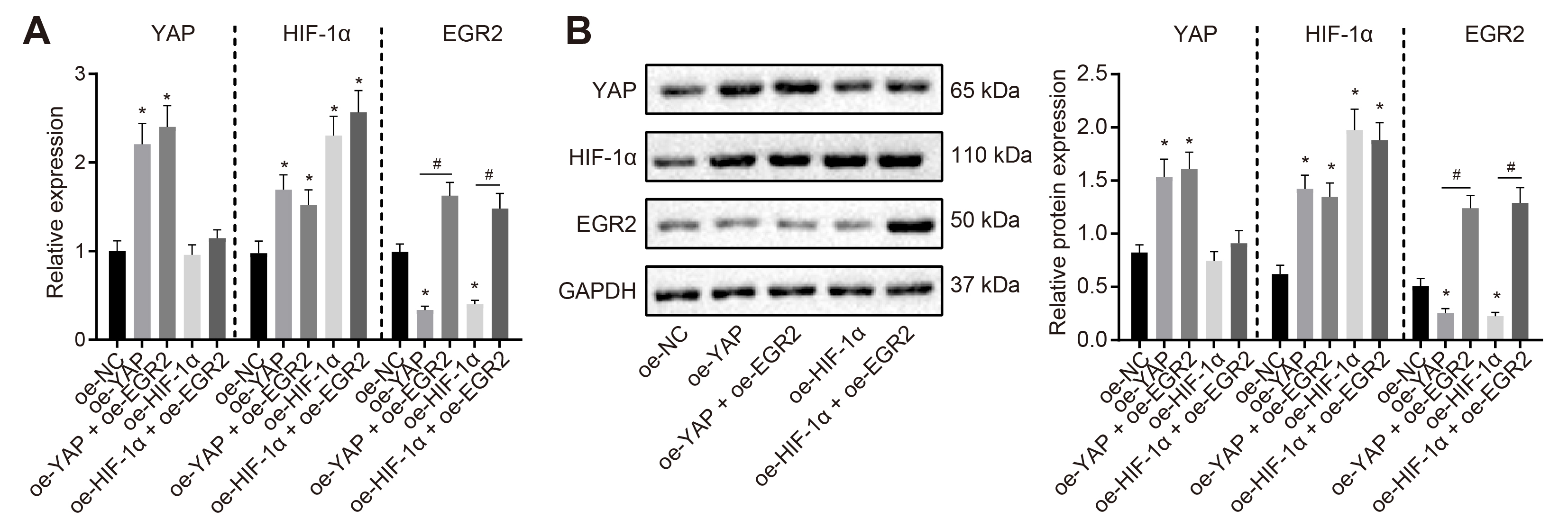

Supplement: Supplementary file 7 — Additional file 7: Figure S7. Expression of YAP, HIF-1α and EGR2 in naive CD4+T cells. A, the mRNA expression of YAP, HIF-1α and EGR2 in cells after different treatments determined by RT-qPCR; B, Western blot analysis of YAP, HIF-1α and EGR2 proteins in cells after different treatments. [file 13578_2021_560_MOESM7_ESM.jpg]

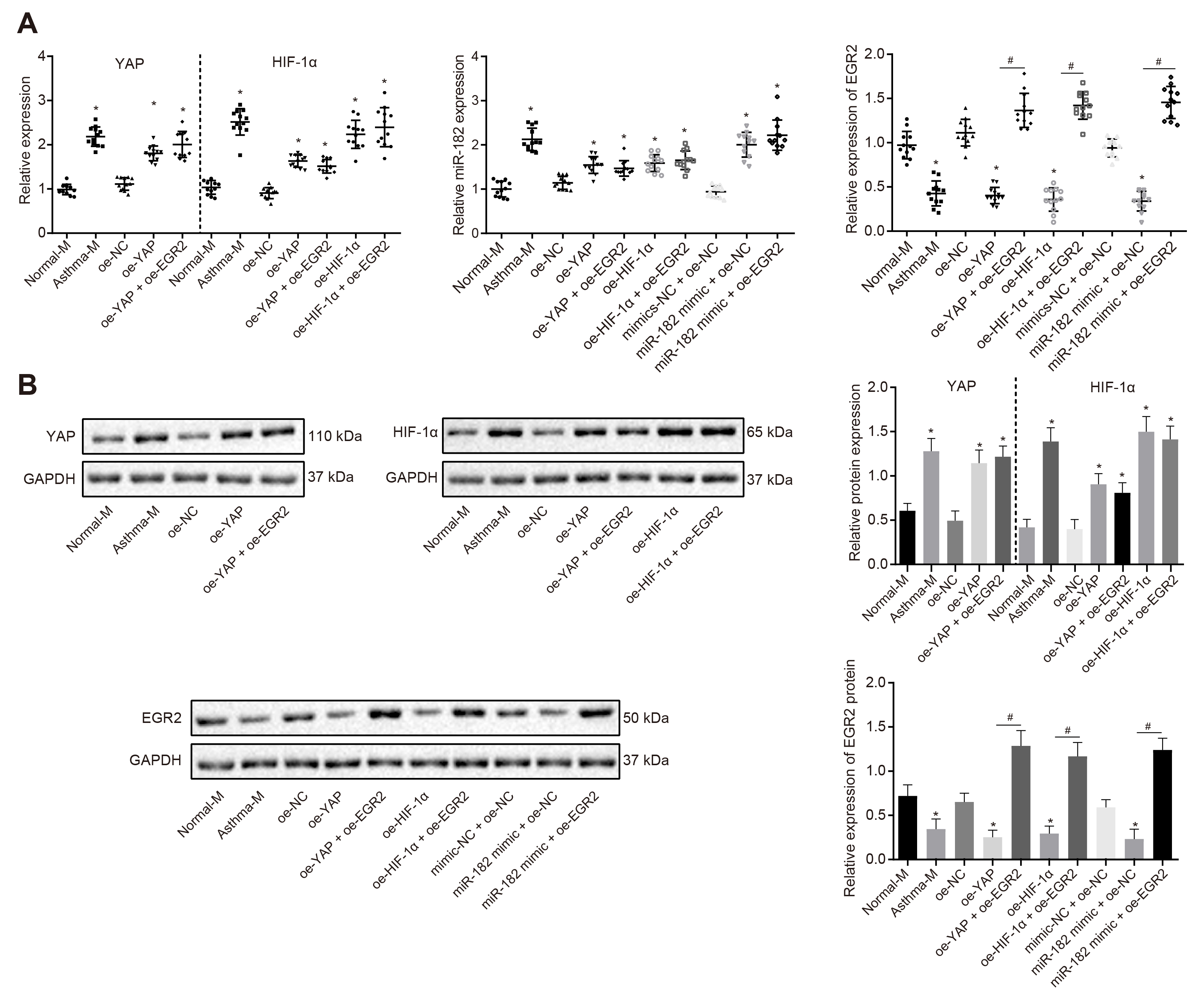

Supplement: Supplementary file 8 — Additional file 8: Figure S8. Expression of YAP, HIF-1α, miR-182 and EGR2 in mouse spleen cells. A, miR-182 expression and the mRNA expression of YAP, HIF-1α, and EGR2 in mouse spleen cells after different treatments determined by RT-qPCR; B, Western blot analysis of YAP, HIF-1α and EGR2 proteins in mouse spleen cells after different treatments. [file 13578_2021_560_MOESM8_ESM.jpg]
